# Plasmid permissiveness of wastewater microbiomes can be predicted from 16S rRNA sequences by machine learning

**DOI:** 10.1093/bioinformatics/btad400

**Published:** 2023-06-22

**Authors:** Danesh Moradigaravand, Liguan Li, Arnaud Dechesne, Joseph Nesme, Roberto de la Cruz, Huda Ahmad, Manuel Banzhaf, Søren J Sørensen, Barth F Smets, Jan-Ulrich Kreft

**Affiliations:** Laboratory of Infectious Disease Epidemiology, KAUST Smart-Health Initiative and Biological and Environmental Science and Engineering (BESE) Division, King Abdullah University of Science and Technology (KAUST), Thuwal 23955-6900, Saudi Arabia; KAUST Computational Bioscience Research Center (CBRC), King Abdullah University of Science and Technology (KAUST), Thuwal 23955-6900, Saudi Arabia; Department of Environmental Engineering, Technical University of Denmark, 2800 Kgs Lyngby, Denmark; Department of Civil Engineering, The University of Hong Kong, Hong Kong, China; Department of Environmental Engineering, Technical University of Denmark, 2800 Kgs Lyngby, Denmark; Department of Biology, University of Copenhagen, 2100 Copenhagen, Denmark; Center for Computational Biology, University of Birmingham, Birmingham, B15 2TT, United Kingdom; Institute of Microbiology and Infection, University of Birmingham, Birmingham, B15 2TT, United Kingdom; School of Biosciences, University of Birmingham, Birmingham, B15 2TT, United Kingdom; Laboratory of Infectious Disease Epidemiology, KAUST Smart-Health Initiative and Biological and Environmental Science and Engineering (BESE) Division, King Abdullah University of Science and Technology (KAUST), Thuwal 23955-6900, Saudi Arabia; KAUST Computational Bioscience Research Center (CBRC), King Abdullah University of Science and Technology (KAUST), Thuwal 23955-6900, Saudi Arabia; Center for Computational Biology, University of Birmingham, Birmingham, B15 2TT, United Kingdom; Institute of Microbiology and Infection, University of Birmingham, Birmingham, B15 2TT, United Kingdom; School of Biosciences, University of Birmingham, Birmingham, B15 2TT, United Kingdom; Department of Biology, University of Copenhagen, 2100 Copenhagen, Denmark; Department of Environmental Engineering, Technical University of Denmark, 2800 Kgs Lyngby, Denmark; Center for Computational Biology, University of Birmingham, Birmingham, B15 2TT, United Kingdom; Institute of Microbiology and Infection, University of Birmingham, Birmingham, B15 2TT, United Kingdom; School of Biosciences, University of Birmingham, Birmingham, B15 2TT, United Kingdom

## Abstract

**Motivation:**

Wastewater treatment plants (WWTPs) harbor a dense and diverse microbial community. They constantly receive antimicrobial residues and resistant strains, and therefore provide conditions for horizontal gene transfer (HGT) of antimicrobial resistance (AMR) determinants. This facilitates the transmission of clinically important genes between, e.g. enteric and environmental bacteria, and *vice versa*. Despite the clinical importance, tools for predicting HGT remain underdeveloped.

**Results:**

In this study, we examined to which extent water cycle microbial community composition, as inferred by partial 16S rRNA gene sequences, can predict plasmid permissiveness, i.e. the ability of cells to receive a plasmid through conjugation, based on data from standardized filter mating assays using fluorescent bio-reporter plasmids. We leveraged a range of machine learning models for predicting the permissiveness for each taxon in the community, representing the range of hosts a plasmid is able to transfer to, for three broad host-range resistance IncP plasmids (pKJK5, pB10, and RP4). Our results indicate that the predicted permissiveness from the best performing model (random forest) showed a moderate-to-strong average correlation of 0.49 for pB10 [95% confidence interval (CI): 0.44–0.55], 0.43 for pKJK5 (0.95% CI: 0.41–0.49), and 0.53 for RP4 (0.95% CI: 0.48–0.57) with the experimental permissiveness in the unseen test dataset. Predictive phylogenetic signals occurred despite the broad host-range nature of these plasmids. Our results provide a framework that contributes to the assessment of the risk of AMR pollution in wastewater systems.

**Availability and implementation:**

The predictive tool is available as an application at https://github.com/DaneshMoradigaravand/PlasmidPerm.

## 1 Introduction

Antimicrobial resistance (AMR) poses a global threat, causing an escalating burden across healthcare settings worldwide (Cosgrove 2006, Antimicrobial Resistance Collaborators 2022). Wastewater treatment plants (WWTPs) serve as key monitoring and control points, connecting various community and hospital sewers with receiving aquatic environments (Cosgrove 2006, Quintela-Baluja *et al.* 2019). WWTPs therefore receive antibiotics originating from human consumption in the community and hospitals (Hocquet *et al.* 2016), which may not diminish even after the treatment process (Kalaiselvi *et al.* 2016) and thus contribute to the residual antimicrobials in the environment (Chang *et al.* 2015). Besides antimicrobials, and probably more importantly, the mixed sewage harbors a diverse array of antimicrobial-resistant strains, which often carry their resistance genes on plasmids. These sites, therefore, serve as hubs in the dissemination network of AMR determinants (Zhang *et al.* 2009, Gatica and Cytryn 2013, Marti *et al.* 2014).

The evolution of AMR is driven by a combination of genetic mechanisms, i.e. mutations and horizontal gene transfer (HGT). Conjugation of plasmids or integrative conjugative elements is a major mechanisms of HGT, which is thought to transfer antimicrobial resistance genes (ARGs) among both closely and distantly related lineages within microbial communities, such as those found in WWTPs (Zhang *et al.* 2009, Halary *et al.* 2010). In WWTPs, commensal and pathogenic strains of human origin are mixed with environmental bacteria, and the high cell density and ability to grow can facilitate genetic exchange of mobile genetic elements carrying ARGs, facilitated by subinhibitory concentrations of residual antimicrobials (Uluseker *et al.* 2021).

The 16S rRNA gene is an essential gene that is conserved across all bacterial and archaeal lineages. However, variation in the hypervariable regions (V1–V9) of the gene allows differential identification of taxa, though only at the genus level when short-read sequencing is used (Sanschagrin and Yergeau 2014, Johnson *et al.* 2019). Despite these limitations, the feasibility and cost-effectiveness of 16S rRNA gene amplicon sequencing has promoted its popularity in microbiome studies. It has allowed detection of associations between taxonomic community composition and various ecological dynamics and habitat characteristics, e.g. disease associations (Gevers *et al.* 2014, Pasolli *et al.* 2016) or ecological status (Cordier *et al.* 2017).

Recent efforts have characterized the potential extent of genetic exchange within WWTPs by leveraging the strengths of 16S rRNA amplicon sequencing and *in vitro* filter mating assays where fluorescent tagging enables the separate collection of transconjugants by fluorescence-activated cell sorting (FACS) followed by sequencing (Actis *et al.* 1999, Musovic *et al.* 2010, Klümper *et al.* 2014, 2015). Using this approach, a recent in-depth analysis of activated sludge microbial communities to serve as recipients of HGT of three broad-host-range multidrug resistance plasmids was carried out (Li *et al*. 2018). The ability of a recipient cell to receive and maintain a given plasmid (at least for a short duration) is referred to as its permissiveness (Li *et al*. 2018). Using the reporter system enables direct quantification of plasmid permissiveness for all recipients in a community, thereby defining the host range of the plasmid within that community. This study did not detect a phylogenetic signal in permissiveness, leading to the conclusion that translating permissiveness from one bacterial group to other phylogenetically similar groups within the WWTP community would not be valid (Li *et al*. 2018). Another study found permissiveness to vary strongly across the taxa of the recipients (Jacquiod *et al.* 2017). However, these studies did not examine the predictive power of sequence markers for plasmid permissiveness.

In the context of microbial molecular ecology studies, machine learning approaches have proven to be effective tools for predicting various phenotypic features, such as AMR, host of isolation, bacterial growth, and virulence, based on genomic biomarkers (Benkwitz-Bedford *et al.* 2021, Aytan-Aktug *et al.* 2022). These models can predict the features, without any prior knowledge about the mechanisms, by learning complex, nonlinear, and high-order phylogenetic signals from labeled sequences in a training dataset. This enables rapid detection of the trait in unseen data (Lupolova *et al.* 2019, Kim *et al.* 2022). Among the various models employed in these studies, ensemble models like random forests and gradient-boosted trees consistently outperformed other models, including linear models. This is because ensemble models combine multiple weak learners to address overfitting while leveraging higher-order interactions between predictive features for more accurate prediction. While several phenotypic features have been used as labels in these models, machine-learning algorithms have not been employed to predict HGT features.

In this study, we leveraged machine learning approaches to assess the degree to which taxonomy (here limited to the V3-4 hypervariable regions of the 16S rDNA) can predict the permissiveness of recipient communities for broad host-range plasmids from *in vitro* permissiveness assays. Such assays require a lot of effort and are thus rarely performed. Predictive power for narrow host-range plasmids should be higher but was not tested as data were not available. We employed various machine learning regression methods for an alignment-free input (i.e. kmer representation) of sequence data. Our results indicate that the sequence data predicted the permissiveness for three broad host-range AMR plasmids with an average accuracy of 0.63 in terms of the correlation between predicted and actual values for unseen data. We identified a set of predictive kmer sequences and how these are distributed across diverse host taxa. These results suggest that permissiveness can be partially predicted based on coarsely resolved taxonomy, without full genome sequencing. This proof-of-concept study demonstrates the applicability of machine learning and lays the groundwork for future studies to predict phenotypic features of HGT from richer metagenomic data.

## 2 Methods

### 2.1 Study design and sampling

To obtain a comprehensive microbiome collection from various time points and locations within the water cycle, we retrieved samples from a WWTP in the UK and another in Denmark during 2017 and 2018. These samples were taken from different locations along the sewage treatment process (Fig. 2A). These sites included residential and hospital sewers, the point where they mix, the WWTP influent, after the primary settlers, in the biological treatment stage, after the secondary settlers and tertiary filters as well as upstream and downstream of the effluent entering the receiving river. Specific sampling dates and locations are provided in Supplementary Table S1.

### 2.2 Experimental filter mating assay

**Figure 1.**
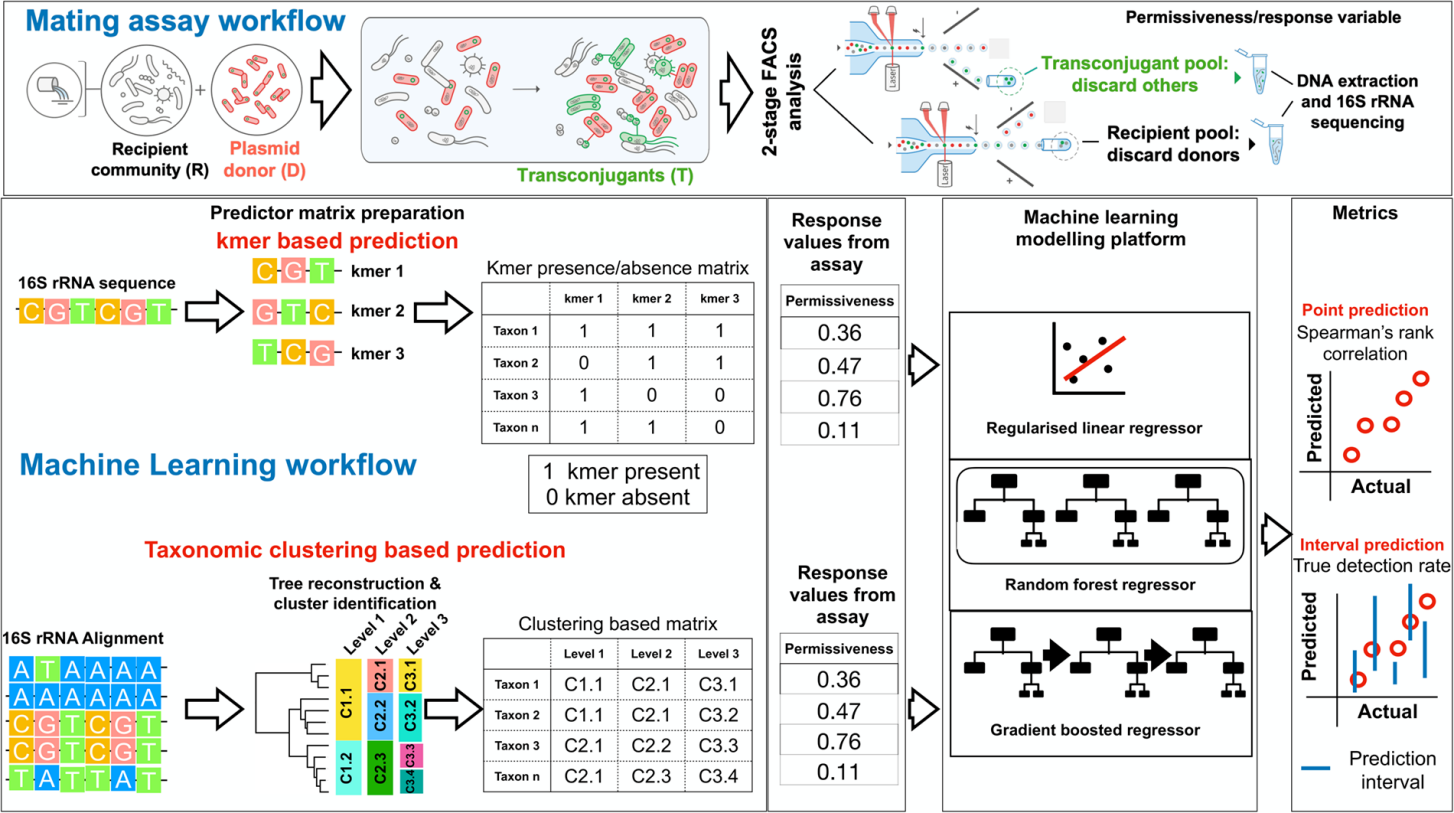
The workflow of the filter mating permissiveness assay and predictive machine learning pipeline. After two-stage FACS separation, first a long sort to collect transconjugants and then a shorter sort to collect all cells but donors (recipient pool), 16S amplicon sequencing was performed separately. For the input of the predictive model, the kmer matrix was converted to a binary matrix, with ones for presence and zeros for absence of the kmer, whereas the group designations for the taxonomic clustering matrix were One Hot encoded.

We employed the solid surface filter mating assay (Musovic *et al.* 2010, Klümper *et al.* 2014) to measure the permissiveness of water cycle microbial communities toward three typical conjugative plasmids (Fig. 1). In brief, the biomass from wastewater was first sonicated to disaggregate sludge flocs but not disintegrate the cells and then left to settle for 5 min, ensuring individual cells remained in suspension. The density of the cells was then adjusted through microscopic cell counting using a Thoma chamber, as described in Klümper *et al.* (2014). These diluted cell suspensions of the WWTP recipient community were then mixed with the donor strain at a 1:1 cell ratio and immediately filtered. The filter was then placed on an agar-solidified synthetic wastewater medium. After incubation (48 h at 25°C) and GFP maturation (48 h at 4°C), transfer events were detected by epifluorescence microscopy. The transfer frequency was quantified as the ratio of conjugation events (CEs), detected as GFP microcolonies, to the original number of recipients (R) in the sample (CE/R). Note recipient cells were untagged bacteria from the environmental sample. We used *Escherichia coli* MG1655 as a donor, which was chromosomally tagged with mCherry expressed from the constitutive promoter pLpp, and carried one of the three plasmids: pKJK5 (IncP-1ε), pB10 (IncP-1β), and RP4 (IncP-1α) (Norberg *et al.* 2011). The plasmids were tagged with pLac-gfp repressed by a chromosomal lacI^q^. Thus, donors exhibited red fluorescence, recipients none, and transconjugants green fluorescence. Supply of the single plasmid in the assays was not limiting as donors were provided in a 1:1 ratio to recipients. Also, donors could grow and donate plasmids repeatedly. According to calculations based on a model of plasmid transfer between bacterial colonies (Lagido *et al.* 2003), nearly all recipient colonies will have grown into contact with growing donor colonies by 14 h of the 48-h permissiveness assay (see Supplementary Text on the modeling of plasmid transfer in filter mating assays). We verified microscopically that at least two-third of the filter area was covered by red-fluorescent donor cells after incubation. Donors on the filter below the biofilm surface would not have been observable. If a recipient carries another plasmid that interferes with the test plasmid, this would render it non-permissive. Plasmids that are long-term residents within a host effectively become part of the host identified by its 16S rRNA sequence.

### 2.3 Sorting and sequencing

For each filter mating, cells were recovered from the filters, and then transconjugants and recipients were gated and sorted by FACS if they were of bacterial size (as detected by the forward scatter) in two stages: In the first longer stage, cells with green fluorescence (transconjugant pool) were collected. In the second shorter stage, cells lacking red fluorescence (“recipient” pool) were collected, as described previously (Klümper *et al.* 2015, 2017). Note this “recipient” pool includes transconjugants, which were originally recipients, and recipients themselves, but not donors. This pool best represents the cells that had the potential to receive the plasmid, compared with the initial wastewater community before mating, i.e. before the few rounds of division that occur on the filter during incubation. Sorted cells were subjected to DNA extraction and PCR amplification of the hypervariable V3–V4 region of the 16S rRNA gene using primer set 341F/806R prior to paired-end sequencing on an Illumina MiSeq platform. We analyzed the paired-end reads of 16S rRNA gene amplicon sequencing using the DADA2 pipeline to obtain amplicon sequence variants (ASVs) (Callahan *et al.* 2016, 2017). We excluded sequences that were longer than 430 bp, resulting in a total of 2272 unique ASVs.

### 2.4 Calculating permissiveness

We define permissiveness as the ability of a host cell (identified as an ASV) to receive a given plasmid and maintain it, at least for a short duration. Permissiveness conflates several successive cellular and molecular subprocesses that collectively result in successful plasmid transfer [e.g. successful interaction between the pilus and recipient cell surface, absence of surface exclusion, absence of restriction or other host immunity functions, etc. (Thomas and Nielsen 2005)]. Yet, unlike these subprocesses, it is measurable for an entire microbial community and it is this overall outcome that is relevant for predicting community-level plasmid dynamics. Permissiveness for broad host-range plasmids is primarily determined by the recipient, but it also depends on the plasmid and donor (Klümper *et al.* 2015, Li *et al*. 2018). Thus, the recipient and the plasmid–donor combination were included in the machine learning, with the recipient represented by its 16S rDNA sequence to quantify the extent to which the 16S signal is predictive. However, the physiological state of the cells, influenced by their current and previous environmental conditions can confound permissiveness results (Klümper *et al.* 2017), which is partially mitigated by standardized assay conditions.

Note that permissiveness is not about plasmid incompatibility, which refers to the inability of two plasmids to stably coexist in the same host and is thus an attribute of the plasmids rather than the host cell. As estimating ASV-specific permissiveness is complicated by the potential growth of both transconjugants and recipients during mating incubation, we calculated apparent permissiveness (AP). It is defined as the ratio of the relative abundance of an ASV in the transconjugant pool to the corresponding recipient pool (Klümper *et al.* 2017). AP thus accounts for the fact that the abundance of an ASV in the transconjugant pool partly depends on its abundance in the recipient pool. When calculating permissiveness, we assigned a count of one to an ASV in the recipient pool if it was absent there while it was present in the transconjugant pool because at least one recipient must have been present if a transconjugant was detected, so the ASV must have been missed, e.g. because the sampling was not sufficiently exhaustive. Permissiveness values reported throughout the article are AP values. The sequences and permissiveness values can be found in Supplementary Table S1. We had two replicates for the pKJK5 plasmid permissiveness measurements taken at the same site. These replicates showed a correlation of 0.74 (Spearman’s rank correlation coefficient) between the values for the same taxa, indicating the repeatability of the measurements.

### 2.5 Machine learning platform

We developed a machine learning platform for predicting permissiveness and identifying predictive sequence signals, see Fig. 1 for an overview. We opted for two approaches: point prediction and interval prediction to account for uncertainty. The point prediction platform invoked a baseline model of regularized lasso linear regression (referred to as lasso) and two ensemble models: gradient boosted regressors and random forest regressors. We used the built-in functions in the sklearn 1.0.2 library for this purpose (Pedregosa *et al.* 2011). We scaled the response variable, permissiveness, prior to feeding it into the machine learning algorithm. We opted for 3-fold cross-validation and split the data into 80% training/validation and 20% test datasets. We tuned the machine learning models using a grid search approach. For the lasso models, we tuned the L2 regularization penalty term by assessing the values 0.0001, 0.001, 0.01, and 0.1. For gradient boosted regressors, we tuned the key parameters: tree depth (1, 3, and 5) and the number of iterations (5, 10, and 30). For random forests, we tuned the key tree-related parameters: number of trees (5, 10, and 30) and tree depth (1, 3, and 5). Manual inspection of some predictions indicated that prediction performance consistently deteriorated with higher numbers of trees and tree depths. We treated kmer length as a parameter and trained three models with different hyperparameter values on them. The selection of the best model for the grid search for each kmer length was based on the highest coefficient of determination regression score function. As shown in Supplementary Fig. S1A, the best-performing models across kmers were attained with certain hyperparameter values for kmer size 5 and a lasso model for pKJK5, and certain hyperparameter values for random forests for RP4 and pB10 (Supplementary Fig. S1A).

To obtain error intervals for the predictions, we repeated training and testing of the models with 10 random train/test data splits. To assess the performance of the tuned models, we computed Spearman’s rank correlation coefficient (Spearman’s *ρ*) between the predicted and actual data instead of Pearson correlation coefficient because of it is suitable for non-normally distributed continuous data and robustness to outliers (Schober *et al.* 2018). The distributions of predicted and actual data (Supplementary Fig. S1B) clearly deviate from normal distributions (*p*-value <.01 from Kolmogorov–Smirnov test for normality) and contain extremely high “outliers” that were not removed from the data as they corresponded to taxa with high permissiveness (they are not “errors”). Note that using Pearson’s *r* as a measure for the accuracy of prediction would not qualitatively affect the findings of the study due to a strong correlation of 0.80 between Spearman’s *ρ* and Pearson’s *r* for the model predictions (Supplementary Fig. S1C).

Besides point predictions of permissiveness, we used random forest models to obtain prediction intervals. These intervals were computed based on the predictions from all the learners (trees) in the final tuned ensemble models (random forests). The intervals account for the uncertainty both in model fitting and in sampling and sequencing. In assessing the model performance, we considered the true detection rate/coverage, which corresponds to the proportion of test observations that were covered by the prediction intervals at different confidence levels. We used the rand_forest() function in R and compared the intervals with the dispersion around the mean (mean absolute difference) and the measurement error range.

We employed an alignment-free approach to generate predictor features (Zielezinski *et al.* 2017). This approach better accommodates highly divergent sequences and allows the trained model to be applied to unseen data without the need for time-consuming multiple alignment and training steps. To this end, we enumerated the kmers of increasing sizes (5, 7, and 9, larger kmer sizes did not improve the prediction performance). This resulted in a matrix indicating the frequency of kmers in each sequence (Fig. 1). We scaled the values using a min–max scaler prior to feeding them to the machine learning models. If a transconjugant’s sequence was found in multiple sites, we averaged the measured AP values for that sequence across the sites. For random forest models, we measured the importance or relevance of features as the decrease in node impurity (the sum of squared residuals) weighted by the probability of reaching that node. The node probability was calculated as the number of samples reaching the node, divided by the total number of samples. The higher the value the more important the feature. To robustly identify important predictive features, we repeated the prediction process using 10 random splits of training and testing data and extracted the important features. We aggregated the results across replicates and retained features that were found to be important across 90% of the replicates in the training dataset. We excluded kmers if they were present in longer predictive kmers and if their presence was not significantly linked with permissiveness (*p*-value from Wilcoxon test < .01). We deployed the trained and tuned models as web and command-line applications, allowing users to estimate the permissiveness for these plasmids for any 16S rRNA sequence they upload. The tool is available at https://github.com/DaneshMoradigaravand/PlasmidPerm.

### 2.6 Taxonomic classification analysis and association analysis

We examined the predictive information contained within the taxonomic clusters inferred from 16S rDNA ASVs of transconjugants for permissiveness, to understand whether elevated/lower permissiveness is linked with particular clades (lineages) or whether it is a trait emerging across multiple taxonomic groups in the transconjugants. Therefore, we employed BAPS, Bayesian Analysis of Population Structure (Cheng *et al.* 2013), as implemented in the R package rhierbaps (Tonkin-Hill *et al.* 2018), to analyze transconjugant communities. Although BAPS was developed to identify population substructures within a single species, we adopted it as a method for identifying clusters at different taxonomic resolutions within 16S rRNA data. We therefore did not need to specify any sequence identity threshold as required for defining OTUs. BAPS robustly identifies partitions of the taxa at various hierarchical levels. This resulted in a membership matrix, which was encoded as a numeric matrix for predictions (Fig. 1). Here, the clusters of ASV features corresponded to panmictic BAPS clusters. We screened increasing numbers of iterations to capture taxonomic classifications at different levels. After identifying the BAPS clusters, the associations with taxonomic groups were then converted into vectors using One Hot encoding before feeding into the machine learning framework. We trained and tuned the models using the same platform as above and assessed the performance of the models on the held-out test dataset. To identify important features (significant predictive lineages/clusters), we repeated the prediction process on 10 random splits of training and test datasets and kept the features that appeared in all replicates. Note that the clusters identified by BAPS as predictive of increased/deceased permissiveness may not always correspond to a single OTU.

To identify the kmers that were not associated with a particular clade, we enumerated all possible kmers with increasing sizes of 5–12 and created a binary input matrix with zeros and ones, indicative of the absence and presence of the kmers, respectively. We then binarized the response variable (plasmid permissiveness) based on its median value. We used the software Scoary (Brynildsrud *et al.* 2016) to examine the association between the kmers and their respective plasmid permissiveness values. Only kmers with Bonferroni-corrected *p*-values smaller than .05, as well as *p*-values corrected for population structure (i.e. the best and worst possible *p-*values reported by the Scoary software) smaller than .05 were kept.

To construct the taxonomic trees, a pairwise distance matrix was created based on the number of kmers shared between pairs of ASVs to generate a neighbour-joining tree using the “ape” package in R (Paradis and Schliep 2019). FigTree (http://tree.bio.ed.ac.uk/software/figtree/) was used to scale the branch length and iTOL (Letunic and Bork 2021) for visualizing the tree and associated annotations.

### 2.7 Evaluation of prediction models with simulated data

We simulated sequences to understand the impact of mutation rate, sample size, and the strength of correlation between predictive kmer and permissiveness on a model’s prediction accuracy and extent of overfitting. We employed Simbac (Brown *et al.* 2016) to simulate collections of 150 bp kmers from 100, 200, 500, and 1000 isolates and under mutation rates of 0.01, 0.5, 1, and 10 mutations per time unit. We introduced a predictive kmer by randomly selecting a kmer with frequency >0.1 20 times. We attributed permissiveness to isolates with and without the kmer by introducing a variable coefficient value (*λ*), which determined the sampling from the baseline pKJK5 plasmid permissiveness. For isolates with the kmer, we drew a random value from the distribution of permissiveness values for the pKJK5 plasmid, truncated between the selection coefficient μ/*λ* and the maximum permissiveness, where μ is the mean of the permissiveness distribution for pKJK5. For isolates lacking the kmer (and thus the given plasmid), we drew permissiveness values from the distribution of permissiveness values for the pKJK5 plasmid truncated between the selection coefficient μ/*λ* and the minimum permissiveness value. Thus, by increasing the absolute values for 1λ, we increased the correlation between the predictive kmer and the permissiveness associated with the kmer. We then fed the simulated data into the predictive random forest pipeline, as detailed above, and computed Spearman’s correlation between the actual and predicted values for the held-out dataset.

## 3 Results

**Figure 2.**
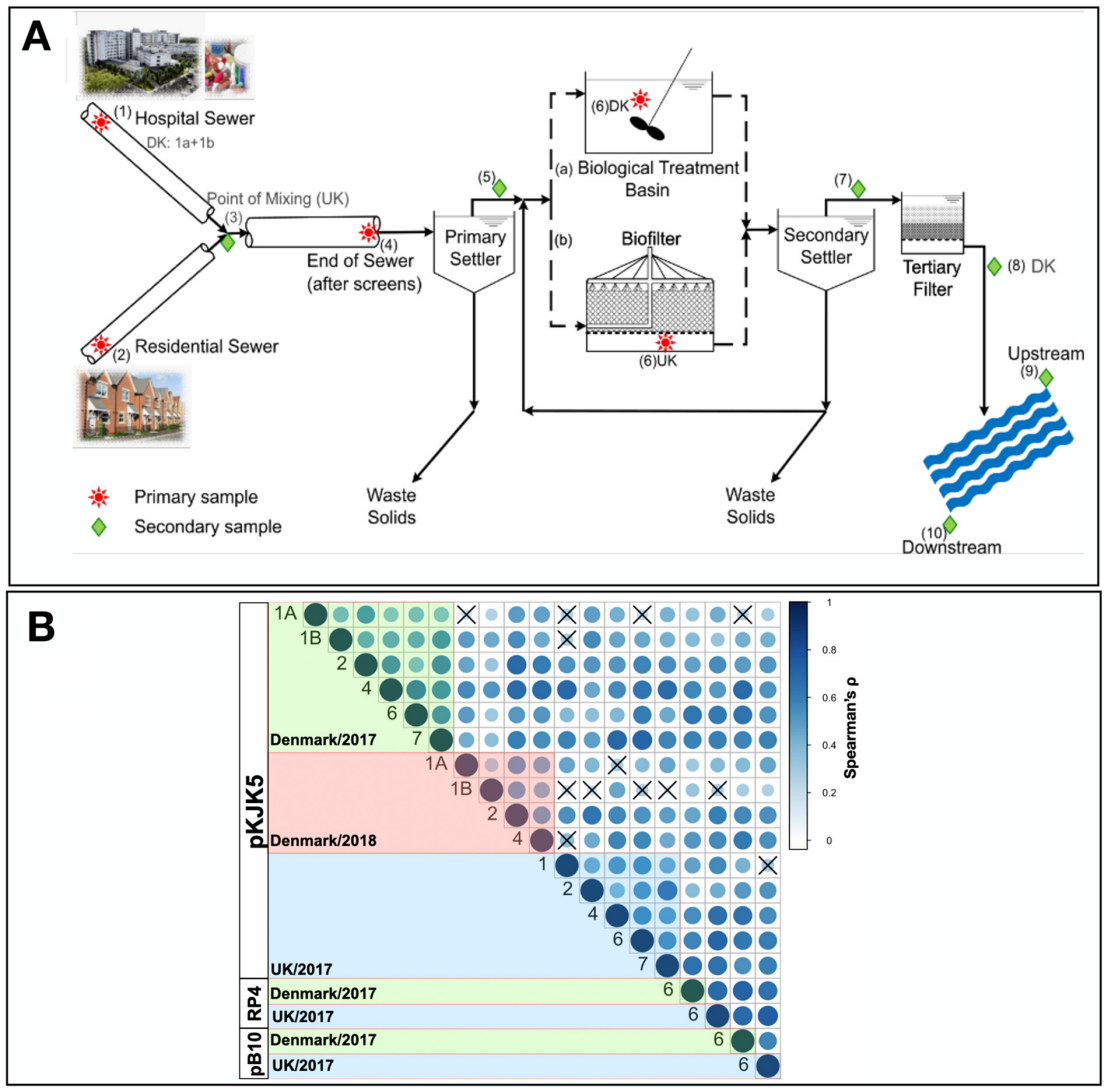
(A) The sampling points across the urban water cycles. Note differences between the WWTPs in the UK using a trickling filter and Denmark (DK) using activated sludge as the biological treatment stage and a tertiary filter. (B) Correlations between plasmid permissiveness values at different sites, dates, and plasmids were all positive. The cross signs label insignificant correlations (*p*-value < .01 from Spearman’s correlation test). The labels on the diagonal axis correspond to the sampling sites in panel (A).

We aimed to understand whether the information in the 16S ASVs can predict plasmid permissiveness, i.e. the ability of cells to receive a plasmid through conjugation (Fig. 1). The permissiveness values were obtained from filter mating assays for samples retrieved in 2017 and 2018 from one WWTP with an activated sludge process in Odense, Denmark, and one WWTP with trickling filters in Durham, UK [detailed in Li *et al.* (2021); Fig. 2A]. Both were receiving residential and hospital sewage. Sampling locations and a schema of the machine learning pipelines are given in Fig. 1.

Overall, plasmid permissiveness showed moderate positive correlations among transconjugant ASVs across various WWTPs, sampling sites, and dates with a mean Spearman’s correlation of 0.49 (range: 0.11–0.73) (Fig. 2B). Except for 13 out of 180 pairwise comparisons of conditions, the correlations were significant (*p*-value < .01, Spearman’s correlation test) (Fig. 2B). These results suggest that the imperfect reproducibility of sampling the same community, fluctuations in community composition over time, the stochastic nature of the mating experiments, as well as experimental errors, limited the achievable correlations: For samples taken from the same site at the same time (replicates for sites 1A and 1B in Fig. 2A) and for samples taken from the same site at different time points in Denmark, average correlations of 0.68 and 0.75, respectively, were found for pKJK5 permissiveness values (Fig. 2B).

Permissiveness values from the UK and Denmark formed distinct clusters (Supplementary Fig. S2A), this may reflect different sewage compositions, environmental conditions, or treatment processes. Despite these country-specific differences, the correlation between permissiveness across different plasmids was high and no clustering of measurements according to the plasmid type was apparent (average Spearman’s correlation of 0.68 across measurements for different plasmids) (Supplementary Fig. S2B and C), suggesting common mechanisms underlie permissiveness in recipient cells. To minimize the impact of time and location of sampling on the performance of the predictive models, we aggregated all permissiveness values for each plasmid.

**Figure 3.**
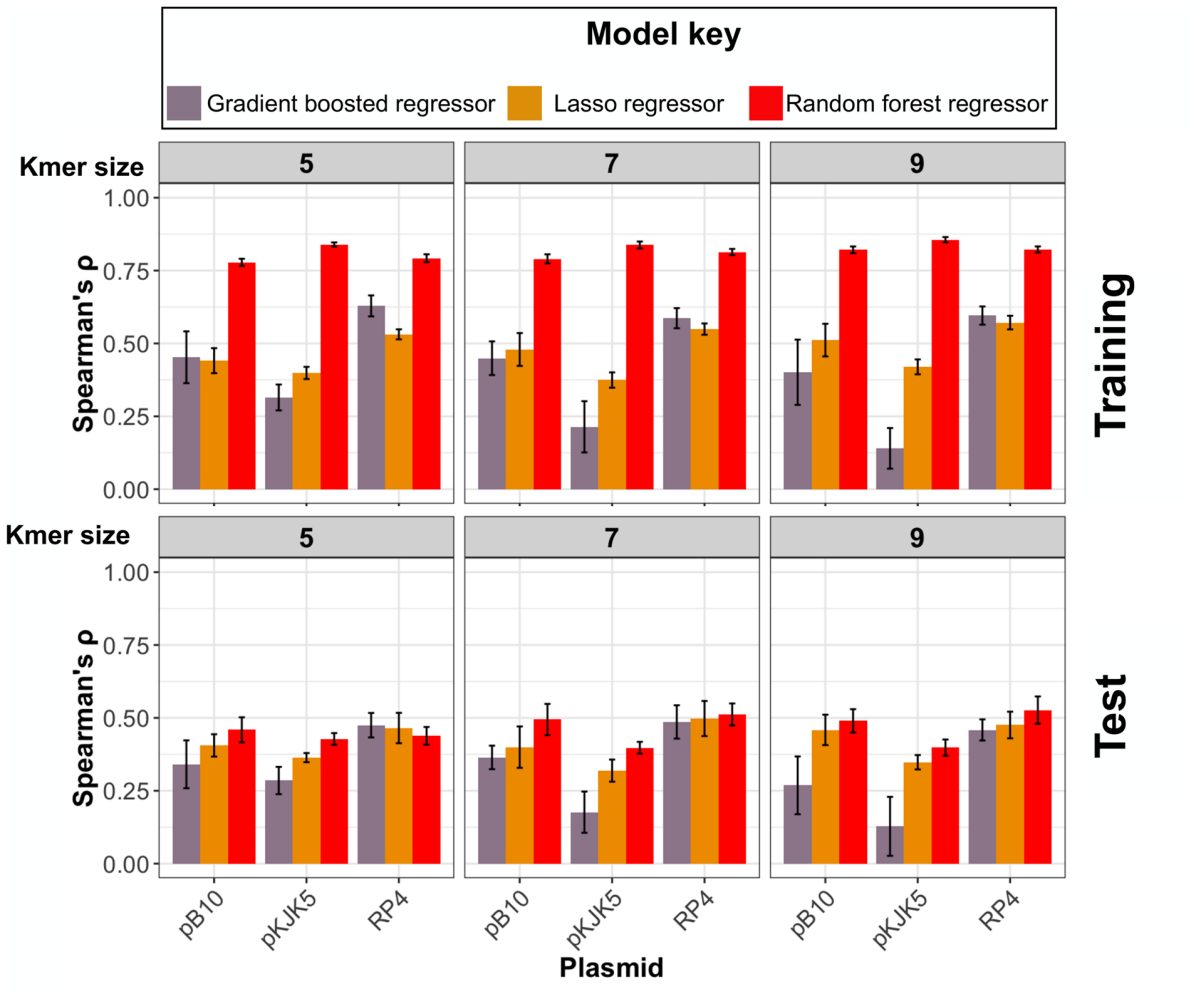
The accuracy of the trained models for predicting plasmid permissiveness for three plasmids (pB10, pKJK5, and RP4) and for gradient boosted, lasso, and random forest regressors, in the training and test dataset, for different kmer lengths. Accuracy was measured as Spearman’s rank correlation coefficient. The error bars show 95% confidence intervals for models trained on 10 random training/test splits.

We fed the permissiveness values for the three plasmids as dependent training data into the predictive models to make point predictions of permissiveness (Fig. 1). The models comprised a baseline regularized lasso regression model, a random forest, and a gradient boosted regressor. They were trained on predictor features, i.e. the counts of particular kmers present (testing one size at a time for different sizes, Fig. 3). The results indicate that the ensemble models, i.e. random forest and gradient boosted regressor models, outperformed the lasso model in 10 out of 12 prediction settings on the held-out dataset, suggesting that accounting for nonlinear interactions improved prediction. Between the ensemble models, the random forest model was superior with the best average accuracy (Spearman’s *ρ* values) of 0.49 for pB10 (95% CI: 0.44–0.55), 0.43 for pKJK5 (95% CI: 0.41–0.49), and 0.53 for RP4 (95% CI: 0.48–0.57) across kmer values (Fig. 3). The extent of overfitting, i.e. the difference between the accuracy for the training and test datasets, did not vary across different models and kmers (Fig. 3). To understand the impact of input transconjugant numbers on mitigating overfitting, we repeated the prediction on down-sampled training datasets (Supplementary Fig. S3). We found that increasing the number of transconjugants steadily improved the accuracy in the test dataset and reduced overfitting, up to a training size of 50% of the full size. Beyond this, the improvement in accuracy leveled off (Supplementary Fig. S3). These findings suggest that a significantly larger training dataset would be required to further improve the prediction accuracy.

**Figure 4.**
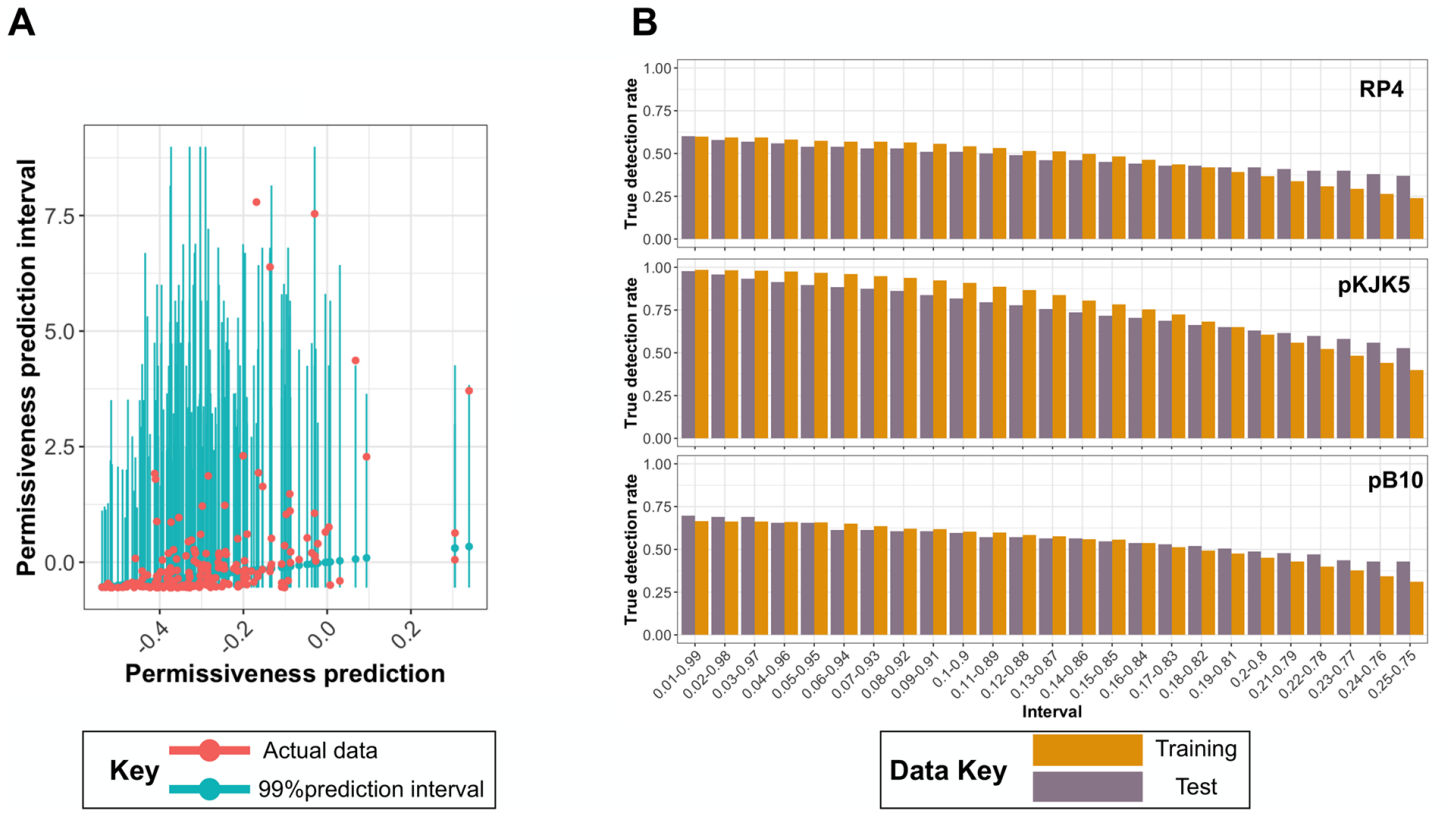
(A) Interval prediction of plasmid permissiveness with the random forest algorithm. Intervals contain 99% of predictions for permissiveness for the pKJK5 plasmid. (B) The true detection rate for shrinking intervals for the pKJK5, RP4, and pB10 plasmids in the training and test datasets. The intervals on the *X*-axis contain the portions of data that fall between the upper and lower limits, e.g. the 0.01–0.99 range includes predictions greater than 1% and smaller than 99% of the values predicted by the model. True detection rate was defined as the relative frequency of data points that fell in the interval. The interval value of 99% corresponds to 0.57×, 2.07×, and 3.5× the dispersion around the mean absolute difference for the permissiveness distributions for pKJK5, pB10, and RP4 plasmids, respectively.

As pointed out above, the differences in permissiveness between two replicates indicated uncertainty from sampling and measurement, which led us to examine the random forest models since they can yield prediction intervals (Fig. 1). We therefore computed the uncertainty associated with random forest model in the form of prediction intervals and then compared them with the measurement error and dispersion. Figure 4A shows the intervals containing 99% of the predictions around the point predictions. For the pKJK5 plasmid and a 99% interval, this is 0.57× and 6× the dispersion around the mean absolute difference and measurement error range, respectively. The trained model achieved a correct detection rate, i.e. the number of times a point prediction is within the prediction interval, of 0.96 for the training and 0.94 for the test dataset (Fig. 4A and B). For the pB10 (RP4) plasmids, the detection rate of the prediction interval was lower and stayed at 0.64 (0.78) for the test dataset at intervals that equaled 2.07× the mean absolute difference and 3.5× the measurement error range. As expected, with narrower prediction intervals, the coverage (detection rate) of the interval steadily decreased; however, the extent of overfitting remained low (Fig. 4B). For pKJK5, with a prediction interval equal to the average measurement error, a detection rate of 0.67 on the test dataset was observed. For prediction intervals that equaled the dispersion of the data around the mean, the intervals included 57% of the permissiveness values for pKJK5 (Fig. 4B). These percentages were 68% and 55% for predicting pB10 and RP4 permissiveness, respectively. Altogether, these results demonstrate the strength of the random forest model in capturing the uncertainty in measurements, which extends its applicability. However, resolving the uncertainty introduced by the model relative to the uncertainty in the experimental results remains an open challenge.

Although conjugation and maintenance of different IncP-1 plasmids is governed by shared mechanisms, genetic divergence in their transfer and regulatory regions has evolved (Norberg *et al.* 2011), which may affect their transfer to, and interaction with, recipient cells, leading to differences in permissiveness. We therefore investigated the generalizability of models trained on one plasmid for predicting the permissiveness for a different plasmid. Prediction accuracy deteriorated for the test data for a different plasmid, when compared with prediction for the same plasmid, with an average drop in correlation (Spearman’s *ρ* for training and test data for the same plasmid−Spearman’s *ρ* for training and test data for a different plasmid) of 0.32 for pB10, 0.14 for pKJK5, and 0.32 for RP4 plasmids (Supplementary Fig. S4A). The partial drop in correlation when predicting permissiveness for other plasmids suggests differences in plasmid interactions with recipient cells.

Like plasmid type, our results indicated that the “country” used for training data affects the accuracy of the prediction but it has to be emphasized that the WWTPs in the UK and Denmark use different biological treatment processes so the “country” effect could be partially an effect of treatment process. Models trained on UK data showed an average decrease in accuracy (Spearman’s *ρ* for training data for both the UK and Denmark and test data for Denmark−Spearman’s *ρ* for training data for the UK and test data for Denmark) of 0.18, 0.15, and 0.19 for plasmids pB10, pKJK5, and RP4, respectively, compared with models trained on mixed data (Supplementary Fig. S4B).

Similarly, when we trained the model on all but one site and assessed the performance on the excluded site, the accuracy decreased by 0.13 on average compared with the accuracy when mixed data were used (Spearman’s *ρ* for training and test data for all sites mixed−Spearman’s *ρ* for training data for all sites except the excluded site and test data for the excluded site) (Supplementary Fig. S4C). Overall, these findings demonstrate the need for representative training datasets to eliminate confounding factors that degrade model performance.

**Figure 5.**
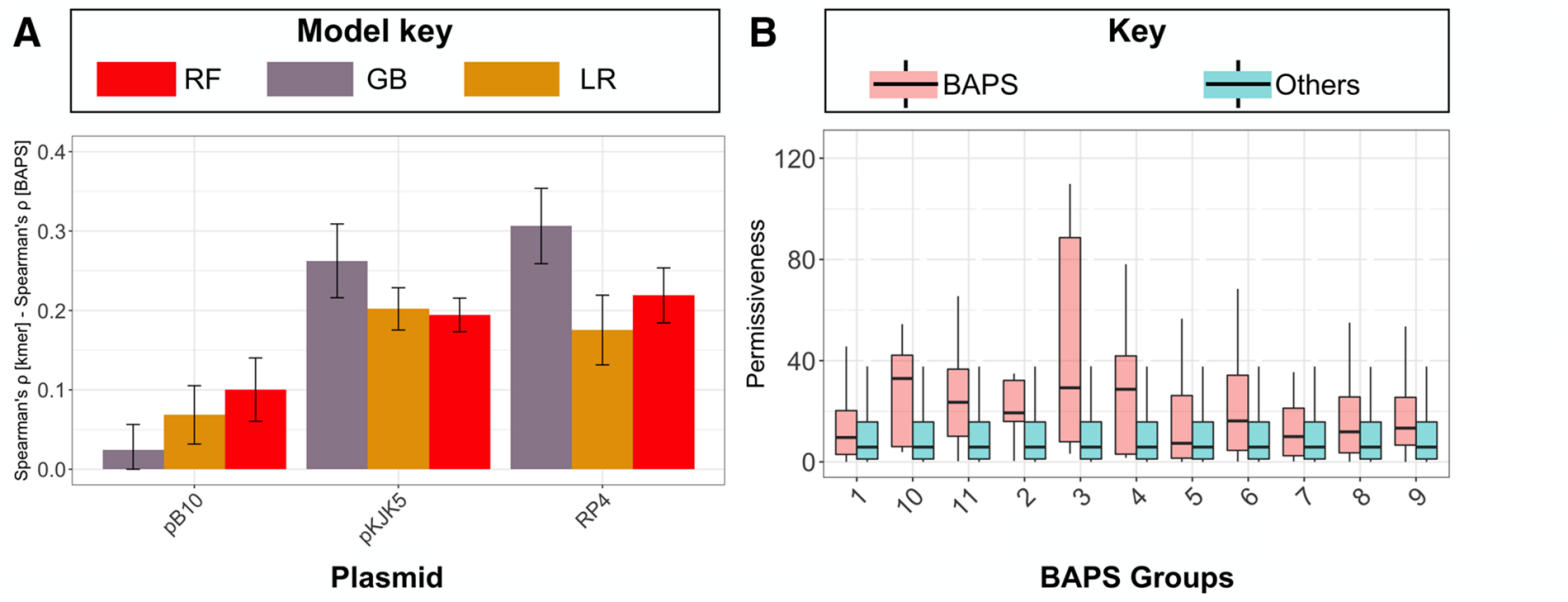
BAPS taxonomic cluster-based prediction. (A) The bars show how much better the kmer-based models were relative to BAPS-based models (bars are the differences between the accuracies of the best performing models for the kmer- minus the BAPS-based models) for different model classes. The terms “RF,” “GB,” and “LR” stand for random forest, gradient boosted, and lasso regressors, respectively. The error bars show 95% confidence intervals across 10 prediction runs with random test/training split. (B) The permissiveness values for ASVs belonging (red) or not-belonging (blue) to predictive BAPS clusters for plasmid pKJK5.

**Figure 6.**
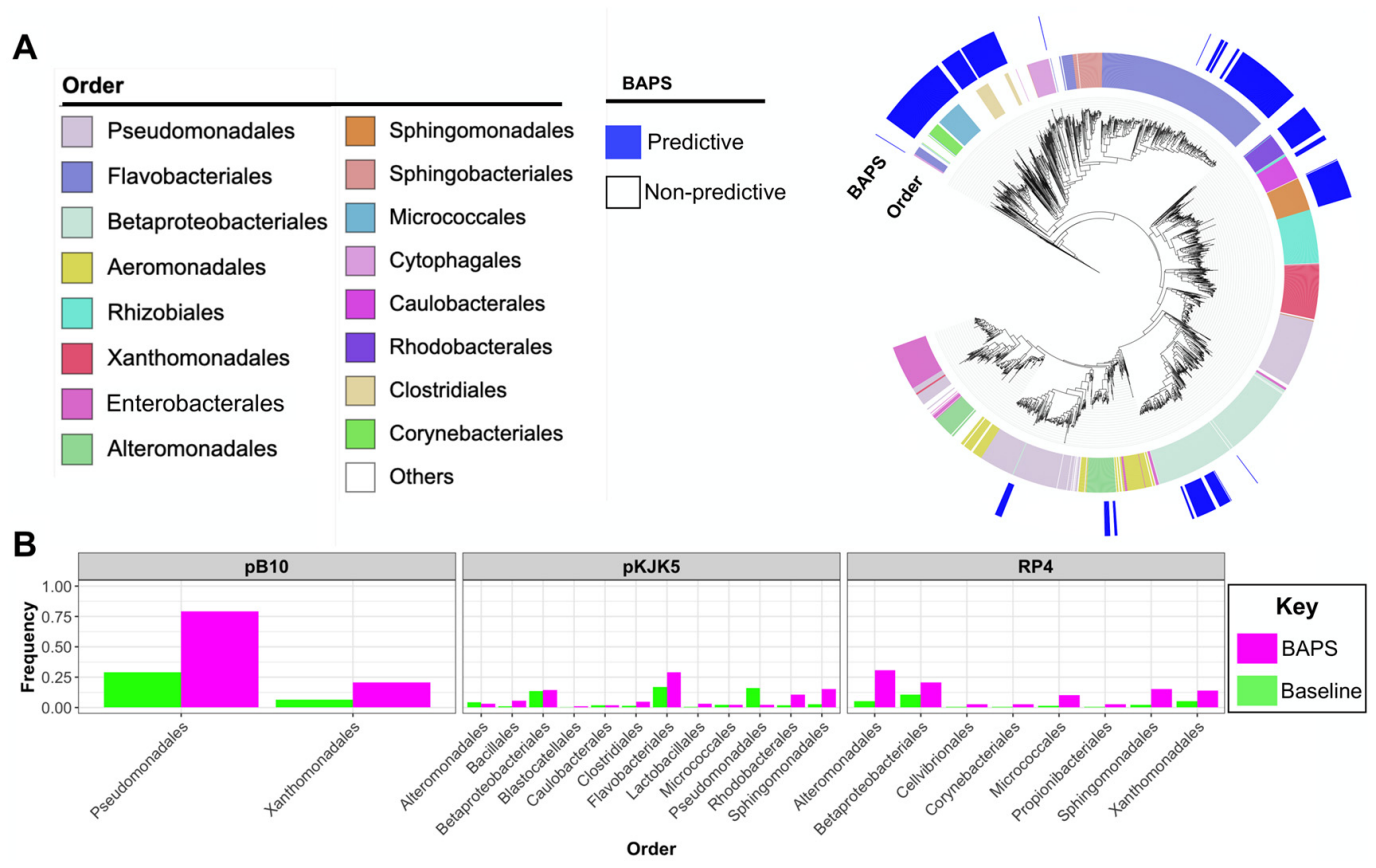
(A) The taxa distribution of predictive BAPS clusters for pKJK5. The clade of unclassified outliers was removed. (B) The frequency of enriched orders in the predictive BAPS clusters for pKJK5, RP4, and pB10 plasmids. The green bars show the baseline frequencies in the recipient pool. We removed 91 taxa that were not annotated from the tree to improve presentation.

We then investigated whether the use of taxonomic clusters can improve predictions of permissiveness. The accuracy of predictions based on clustering ASV features (panmictic BAPS clusters) turned out to be consistently lower than predictions based on kmers (Fig. 5A). For different types of models, those trained on BAPS cluster information had an average drop in accuracy of 0.06 for pB10, 0.23 for RP4, and 0.21 for pKJK5, when compared with the best performing kmer-based prediction (Fig. 4A). Nevertheless, we identified eight predictive BAPS clusters for pKJK5, which were positively linked with high permissiveness values (*p*-value from Wilcoxon test < .01) (Fig. 5B). The phylogenetic distribution of these BAPS clusters showed that they occur across a wide range of taxa (Fig. 6A). We compared the frequencies of taxa contained in predictive BAPS clusters with the baseline frequency of the same taxa to identify the enriched taxa (Fig. 6B). Previous studies reported a broad host range for the pKJK5 plasmid (Musovic *et al.* 2006, Shintani *et al.* 2014, Klümper *et al.* 2015). In line with these reports, the predictive BAPS clusters encompassed both Gram-negative and Gram-positive orders, with notable overrepresentation of Sphingomonadales and Bacillales. This finding highlights the importance of HGT in the evolution of these strains, as shown for phage-mediated gene transfer (Soffer *et al.* 2015). The Gram-positive significant orders included a wide range of orders, e.g. Clostridiales, Bacillales, Micrococcales, and Corynebacteriales (Fig. 6B). The enriched orders for pB10 and RP4 were like those for pKJK5 but with some unique orders. While we observed a sharing of predominant orders for pKJK5 with pB10 and RP4 plasmids, e.g. Micrococcales and Sphingomonadales, some recipients’ orders, e.g. the Proteobacterial orders Xanthomonadales, Alteromonadales, and Pseudomonadales (Fig. 6B), appeared exclusive to pB10 and RP4 plasmids. These orders contain many pathogens, in which HGT plays a major role in driving the evolution of adaptation, AMR, and pathogenicity in humans and plants (Caro-Quintero and Konstantinidis 2015, Chen *et al.* 2018). Altogether, these findings suggest the existence of plasmid-specific interactions and shared recipient features within certain taxonomic clusters.

**Figure 7.**
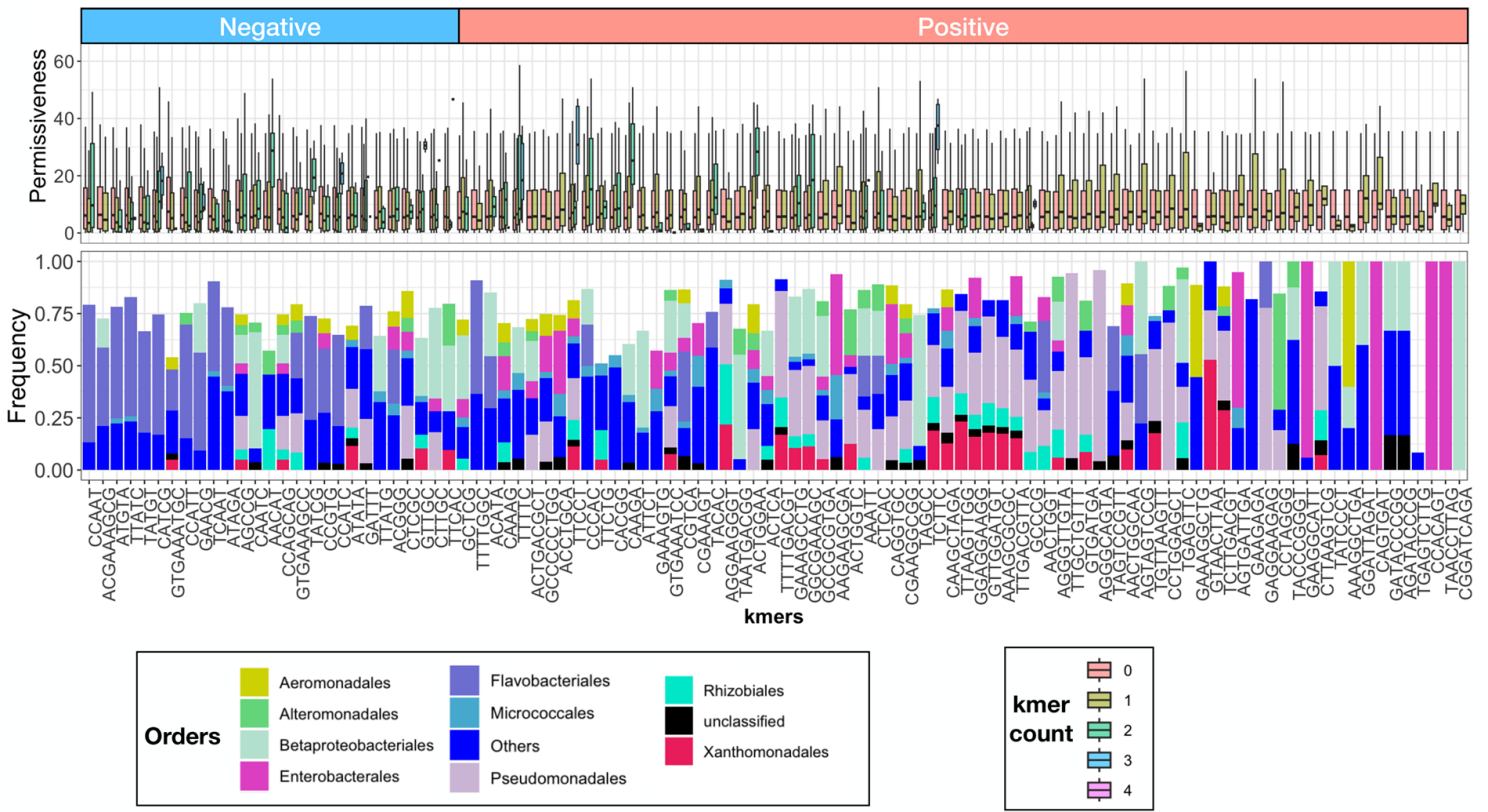
Feature importance analysis for kmer-based predictions. For the top hundred predictive kmers (based on their predictive power ranking) for pKJK5, the relative frequency of taxa with the respective kmer as well as the correlation of the presence of the different counts of the concerned kmers with plasmid permissiveness is shown. The box plots show the distribution of permissiveness values for ASVs (taxa) bearing the kmer. The groups of box plots are sorted by increasing difference between the mean permissiveness for ASVs with versus without the kmer. The “negative” group of boxplots shows kmers whose absence is linked with increased permissiveness, while the “positive” group of boxplots shows kmers whose presence is linked with increased permissiveness. The bar plots show orders that were enriched in the group of ASVs harboring the kmers compared with the baseline distribution.

To examine the predictive information contained in kmers, we next identified the kmers most predictive for permissiveness. The feature importance analysis for significant kmers pinpointed 661, 146, and 71 kmers, of which 371, 89, and 65 were positively linked with permissiveness for pKJK5, pB10, and RP4, respectively (Fig. 7 and Supplementary Figs S5 and S6). Like the BAPS clusters, we observed a distinctive distribution of significant kmers for each plasmid. However, we observed a higher discriminatory power between ASVs with and without the predictive kmers, compared with ASVs from the predictive and non-predictive BAPS clusters (Supplementary Fig. S7). This implies that information in the predictive kmers captured a greater variance in the data, when compared with information in predictive BAPS clusters, pointing to multiple taxonomic signals within the ASVs that only kmer-based predictions used. This result is congruent with the better performance of kmer-based predictions. The predictive kmers for the plasmids were weakly correlated and covered a wide range of orders, some of which were identified by BAPS-based analysis (Fig. 7 for pKJK5 and Supplementary Fig. S4 for pB10 and RP4 plasmids). For pKJK5, the kmers linked with the Gram-negative orders of Enterobacterales, Betaproteobacteriales, Pseudomonadales, and Xanthomonadales most strongly predicted permissiveness (Fig. 7). For RP4 and pB10, besides Pseudomonadales and Cellvibrionales, which were identified by BAPS analysis, Aeromonadales ASVs appeared to contain predictive kmers (Supplementary Figs S5 and S6), which aligns with recent evidence of HGT in the *Aeromonas* genus in aquatic environments (Bello-López *et al.* 2019). Predictive kmers, whose absence was linked with increased permissiveness, showed somewhat similar distributions for plasmids: For RP4 and PB10, these kmers occurred in Flavobacteria, which consistently showed a lower permissiveness for these plasmids (Supplementary Fig. S6). For pKJK5, the signals also occurred throughout various clades, besides Flavobacteria (Fig. 7). Altogether, the kmer analysis appeared to identify further predictive signals for permissiveness.

The lower performance of the models based on taxonomic clusters, when compared with kmer predictions, also suggested that elevated permissiveness may have lineage independent signals. To identify these signals, we screened the kmers for association with permissiveness after accounting for lineage associations. In total, we filtered 8, 6, and 5 significant kmers with unique distributions for pKJK5, RP4, and pB10 plasmids linked with elevated permissiveness, respectively (Supplementary Fig. S8). Like the BAPS and kmer results, the distribution of significant kmers differed between the plasmids (Supplementary Fig. S8). For pB10 and RP4, the kmers were predominantly found within Gram-negative clades of Pseudomonadales, Aeromonadales, and Cellvibrionales and to a lesser extent in Gram-positive Corynebacteriales strains. For Corynebacteriales strains, HGT is recognized to contribute to their pathogenicity and AMR (Zhi *et al.* 2017). The significant kmers for pKJK5 were found across a wider range of Gram-negative and Gram-positive species (Supplementary Fig. S8). The presence of these host sequence signals in only very distantly related taxa suggests a convergent evolution of molecular mechanisms for transfer and maintenance of IncP-1 plasmids in Gram-positive and Gram-negative strains (Goessweiner-Mohr *et al.* 2013), which may be discovered by whole genome data analysis.

We then examined the extent to which the number of 16S rRNA sequences used for training and a given mutation rate would affect the performance of the predictive models. Henceforth, we carried out predictions with simulated 16S rRNA data with various values for the strength of correlation between the predictive kmer and permissiveness, mutation rate, and population size (Supplementary Fig. S9). The results indicate that for a wide range of parameter values for population size and correlation of the predictive kmer with permissiveness, the prediction accuracy for the test dataset remained between 0.65 and 0.75 (Supplementary Fig. S9B). With increasing population size, the accuracy of the trained model for the test data did not seem to improve, implying the existence of limits for correct predictions. The extent of overfitting, i.e. the difference in accuracy between test and training data, increased with higher mutation rates (Supplementary Fig. S9A). This observation may be explained by the high clonality of populations at low mutation rates, which makes it likely that a taxon from the test dataset falls within the same clade as the training dataset, thus making the training data highly predictive of the test data. With increasing mutation rate, although a larger number of predictive signals are available, which improves the accuracy of prediction in the training dataset, the generalizability of the model to the test data declines because of a higher divergence between input sequences in training and test datasets. Despite these limitations, the results indicate that the models achieve a high accuracy across a wide range of parameter values.

## 4 Discussion

While it is expected that permissiveness for narrow host-range plasmids should have a clear taxonomic signal that facilitates prediction, this was not clear for broad host-range plasmids. Moreover, there is significant microdiversity (Jaspers and Overmann 2004) between strains with the same full-length 16S rRNA sequence and even more so with partial sequences used here, which could make predicting permissiveness rather hopeless. Nevertheless, we demonstrated that prediction is possible yet limited. We present a machine learning framework for predicting plasmid permissiveness from 16S rDNA amplicon sequencing (V3–V4 hypervariable regions) of recipient and transconjugant pools from filter mating assays, inoculated with samples from various compartments of the urban water cycle. Despite the short length of the predictor sequence, our results show that the genetic information of the host captured in 16S rRNA sequences may account for around 50% of the total variance in permissiveness for different resistance plasmids. Furthermore, our analysis identified predictive biomarkers for permissiveness of recipient cells and provided evidence for host preferences and plasmid specificity across multiple lineages.

Although our results with IncP-1 plasmids provide evidence for lineage specificity of successful HGT even for broad host-range plasmids, this specificity depended on the plasmid type. However, we also found a broad phylogenetic distribution of elevated permissiveness, with molecular signals spanning multiple divergent clades. Permissiveness presumably requires suitability of the host for establishing transfer, avoiding entry exclusion and host immunity systems, e.g. restriction–modification and CRISPR-Cas systems (Price *et al.* 2016, Benz *et al.* 2021), and compatibility with the host and any resident plasmids (San Millan and MacLean 2017). Permissiveness also requires the expression of plasmid genes to replicate and partition the plasmid, at least for a few divisions.

Several studies reported specific genetic conflicts between chromosomal and plasmid genes that lead to high fitness costs for the host that could be mitigated by compensatory mutations in either the host chromosome, the plasmid, or both (Dahlberg and Chao 2003, Dionisio *et al.* 2005, De Gelder *et al.* 2007, Harrison *et al.* 2015, San Millan *et al.* 2015, Loftie-Eaton *et al.* 2017, Hall *et al.* 2021). This study identified hubs for conjugation that may consist of certain lineages already harboring suitable compensatory mutations, thus providing predictive genomic signals for permissiveness. While the taxonomic distribution of these mutations in full genomes is yet to be elucidated, the elevated permissiveness across distantly related taxa points to possible convergent evolution for plasmid uptake and carriage within hosts.

The unexplained variance in the output of the models likely has various causes. The physiology of the host cell at the time of the experiment will depend not only on its genome (its potential physiology), which is not fully represented by its 16S rRNA phylotype, but also on its transcriptome, proteome, and metabolome (its current physiology). The latter will depend on the environmental conditions experienced over the past few generations while the host cell was being transported through several water cycle compartments or resided in one particular compartment with longer solid residence times. The filter mating conditions can also affect cellular activities and shift the community composition, e.g. the synthetic wastewater medium differs from the actual water sampled. Therefore, the experimental results may not directly apply to *in situ* plasmid permissiveness. However, standardized test conditions are essential to eliminate environmental confounders. Certainly, seasonal, diurnal, and higher frequency temporal fluctuations combined with considerable spatial heterogeneity will affect the microbial community in the various compartments of the water cycle sampled (McLellan *et al.* 2010). To minimize the effect of the environmental heterogeneity and host cell diversity on the performance of the predictive models, a more complete, representative, and balanced training collection is essential. An ideal sampling framework would include sufficient samples to represent temporal and spatial variation across all compartments, including the different types of wastewater treatment processes, as has been done for the microorganisms found in WWTPs by the MIDAS consortium (Dueholm *et al.* 2022). Such a collection would maximize the viability and generality of the trained models.

While we identified elevated permissiveness in taxa previously reported to have high permissiveness, some taxa, e.g. *E.coli*, did not appear as permissive in our study as commonly reported. The low permissiveness we observed for *E.coli* might be caused by the conditions during the mating (synthetic wastewater medium, 25°C) that are suboptimal for *E.coli*. In addition, the *gfp* expression from our plasmids is repressed by the product of *lacI*. This gene is typically present in *E.coli*, so it is possible that permissiveness was underestimated for taxa with high *lacI* expression.

Several previous studies have attempted to predict complex bacterial traits from full genomic data of naturally occurring strains. The traits included environmental niches, host phenotypes, host specialism, AMR, and bacterial growth features (Wheeler *et al.* 2018, Asgari *et al.* 2019, Benkwitz-Bedford *et al.* 2021). However, no machine learning model has been developed for HGT. The predictive power of partial 16S rRNA gene sequences with signatures of lineage dependence that we report here sets the stage for future research, where full genome sequences or metagenomic features are used as further predictor signals for the models and feature importance analysis. The models developed here and future models can serve as useful tools for assessing the potential risk of mixing different wastewaters containing both resistant and sensitive bacterial strains or releasing these waters into receiving aquatic habitats, enabling subsequent resistance transmission across the environmental systems. Tools with such predictive capabilities can play a major role in supporting One Health efforts. Such models, trained on data from studies aiming to reduce plasmid transfer in laboratory settings (Buckner *et al.* 2020), can enable prediction of the effect of these interventions on a wide range of microbial communities in different environments to assess the utility of these interventions in relevant clinical and non-clinical settings.

## Supplementary Material

btad400_Supplementary_Data
