# Supplementary material for: Plasmid permissiveness of wastewater microbiomes can be predicted from 16S rRNA sequences by machine learning
Source: Bioinformatics. 2023 Jun 22;39(7):btad400. doi: 10.1093/bioinformatics/btad400 (PMC10318386; doi:10.1093/bioinformatics/btad400)
Supplement: btad400_Supplementary_Data [file btad400_supplementary_data.zip › SupplementaryMaterial(Figures&Text).pdf]

Supplementary Material for

Plasmid Permissiveness of Wastewater Microbiomes can be Predicted from 16S rRNA Sequences by Machine Learning

Danesh Moradigaravand[1,2]*
Liguan Li[4,5]
Arnaud Dechesne[4]
Joseph Nesme[6]
Roberto de la Cruz[3,7,8]
Huda Ahmad[3]
Manuel Banzhaf[7,8]
Søren J Sørensen[6]
Barth F Smets[4]
Jan-Ulrich Kreft[3,7,8]*

1−KAUST Smart-Health Initiative and Biological and Environmental Science and Engineering (BESE) Division, King Abdullah University of Science and Technology (KAUST), Thuwal 23955-6900, Kingdom of Saudi Arabia
2−KAUST Computational Bioscience Research Center (CBRC), King Abdullah University of Science and Technology (KAUST), Thuwal 23955-6900, Kingdom of Saudi Arabia
3−Center for Computational Biology, University of Birmingham, UK
4 −Department of Environmental Engineering, Technical University of Denmark, Lyngby, Denmark
5 −Department of Civil Engineering, The University of Hong Kong, Hong Kong, China
6 −Department of Biology, University of Copenhagen, 2100 Copenhagen, Denmark
7−Institute of Microbiology and Infection, University of Birmingham, Birmingham, UK
8−School of Biosciences, University of Birmingham, Birmingham, UK

Published in Bioinformatics in 2023

Contents:

Supplementary Figures

Supplemental Text: Modeling of plasmid transfer in filter mating assays to assess potential plasmid donor limitations to completeness of transfer

Supplementary Figures

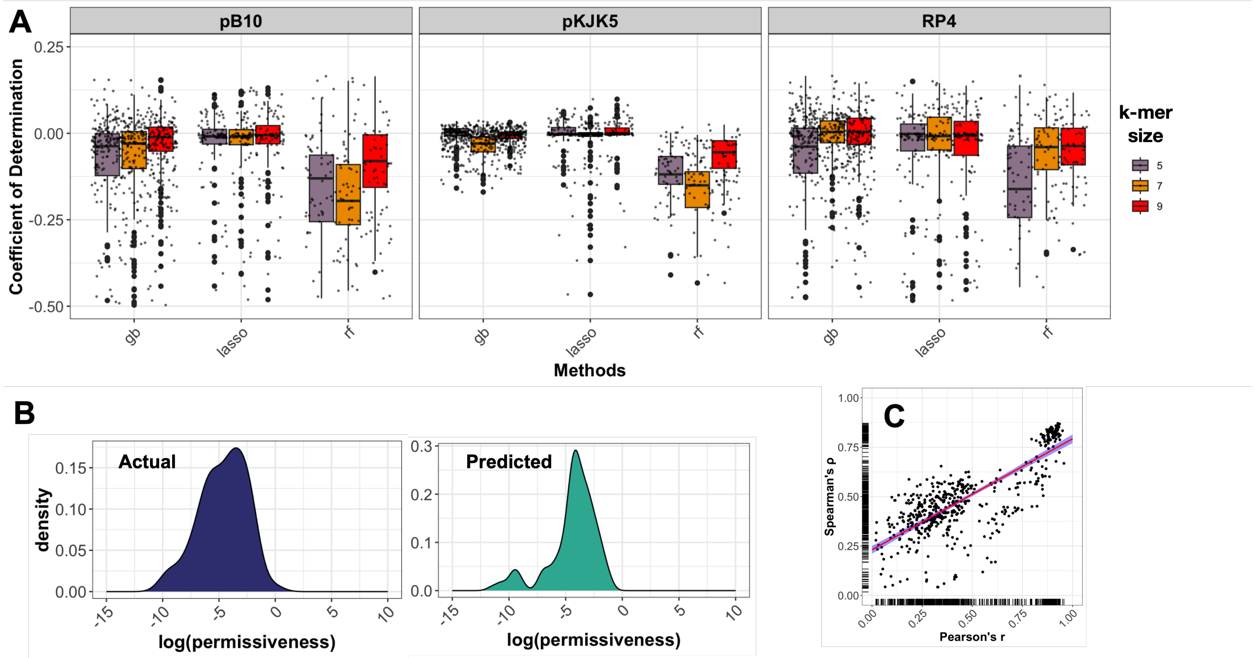

Supplementary Figure S1. Performance metrics for the models. **(A)** The distribution of the coefficient of determination values (www.scikit-learn.org/stable/modules/generated/sklearn.metrics.r2_score.html) used for choosing the best performing model in the grid search for different kmers. Each dot shows one set of hyper-parameter values. The terms "rf" and "gb" refer to random forest and gradient boosted regressors, respectively. The best performing models, i.e. models with the highest coefficient of determination values on the training/validation dataset, were attained for kmer size 9 and random forests for pB10, kmer 9 and lasso regressor for pKJK5 and the kmer 5 and random forests for the RP4 plasmid. **(B)** The distribution of predicted and actual permissiveness values for a sample run with kmer size 5 and random forest model for a test data set for pB10 plasmid. **(C)** The correlation between Pearson's r and Spearman's ρ for the predictions on both test and train datasets. The red line and the blue ribbon show the fitted line and 95% confidence interval. The correlation between the two correlations was 0.80 (Pearson's r).

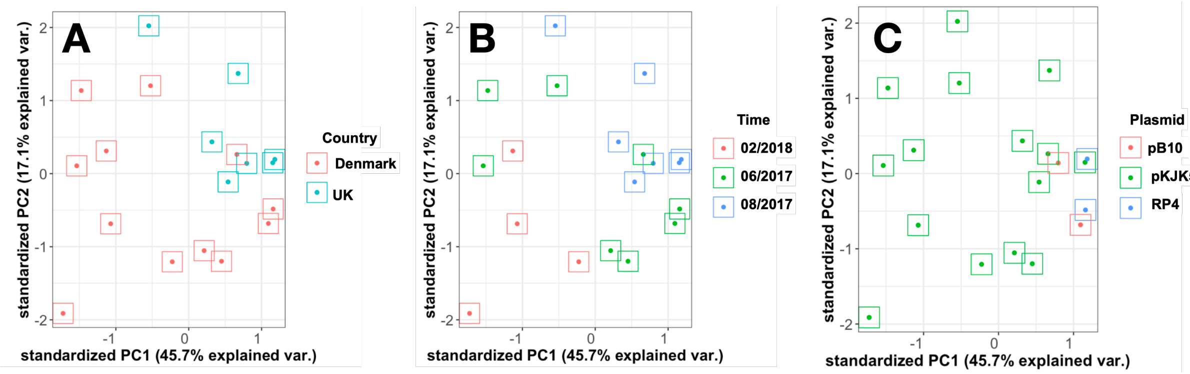

Supplementary Figure S2. PCA plot for the measured permissiveness values colored according to **(A)** 'country', **(B)** time of sampling (month and year) and **(C)** plasmid. Note there are many differences between the 'countries', including different treatment processes (trickling filter in the UK and activated sludge in Denmark).

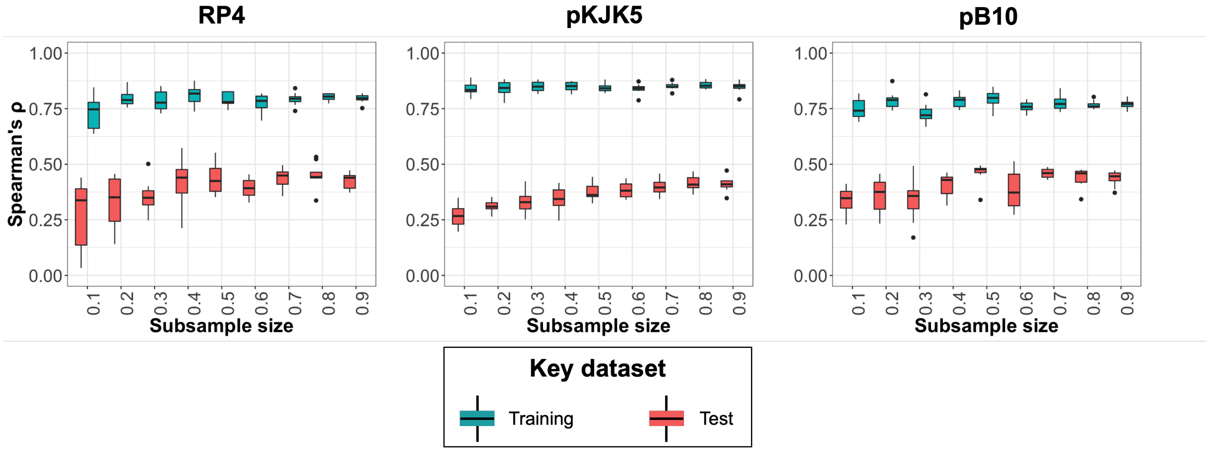

Supplementary Figure S3. The effect of sample size (transconjugant numbers) on the accuracy of prediction and the extent of over-fitting. Bluegreen and red boxes correspond to the accuracy of prediction for 10 randomly drawn sub-samples for the test and training datasets, respectively. We trained and tuned a random forest model with kmer size of five.

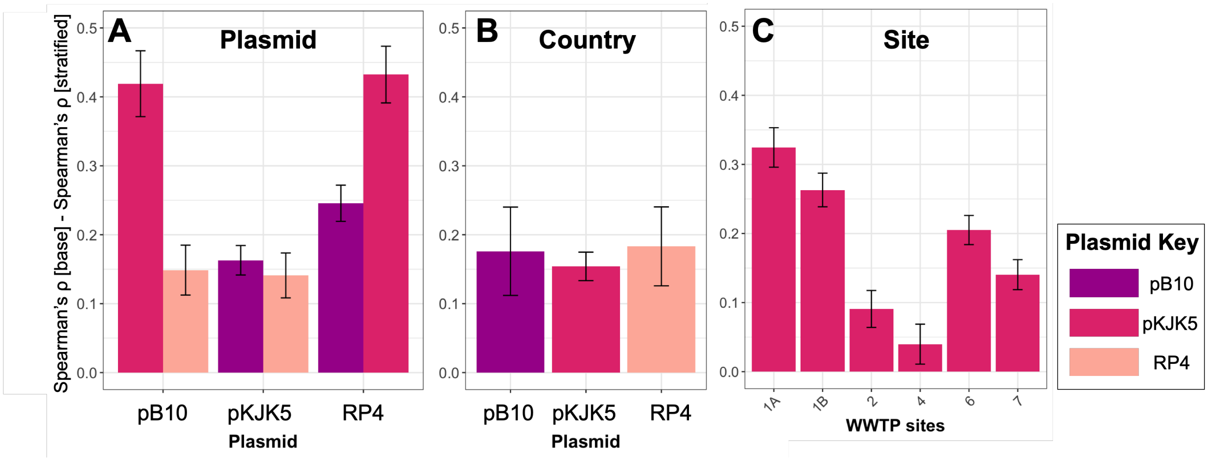

Supplementary Figure S4. Differences between the accuracy of the baseline model and models trained or tested on stratified training datasets. Higher bars indicate a more significant effect of training sample composition (stratified by plasmid, country of sampling, and site) on prediction accuracy. **(A)** The bars show the difference between the accuracy for the baseline model and models trained on data for the focal plasmid, as indicated on the x axis, that were then used to predict the permissiveness for the two other plasmids, as indicated by the color key. Here the baseline is the model trained and tested on the dataset for the focal plasmid. **(B)** The bars show the difference between the baseline model and models trained on UK data, which were tested on data from Denmark. The baseline model is the model trained on data from both UK and Denmark and tested on data from Denmark. **(C)** The bars show the difference between the baseline model and models trained on pKJK5 permissiveness values for transconjugants from all sites, except the site indicated on the axis that was used for testing. The baseline model is the model trained on data that consisted of transconjugants from all sites. Numbers on the x-axis denote the sites in Figure 2A. The error bars show 95% confidence intervals for ten random training/test data splits.

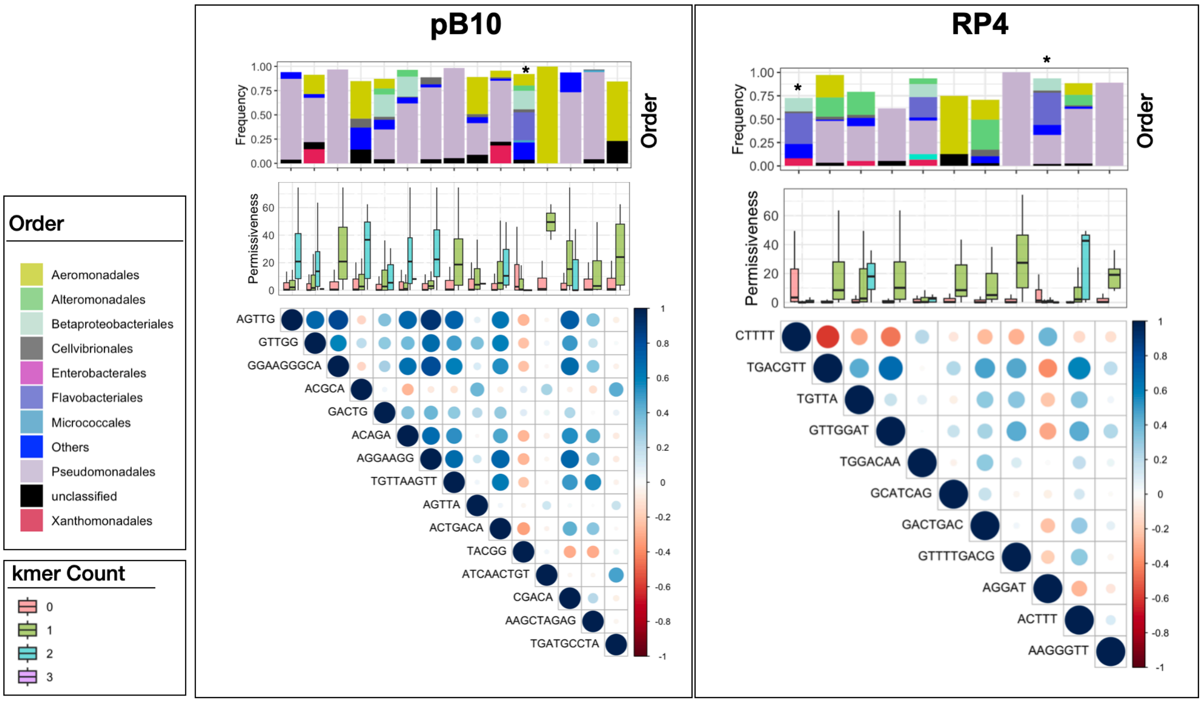

Supplementary Figure S5. Top 15 and 11 predictive kmers for **(A)** RP4 and **(B)** pB10 plasmid permissiveness, respectively, based on their predictive power ranking. The correlogram shows the correlation between the presence/absence pattern of the kmers across taxa. The boxplots show the distribution of permissiveness for the plasmids in taxa with different numbers of kmers. The bar plots show the relative frequency of orders containing predictive kmers. To improve presentation, only the results for the top ten percent of the kmers, based on their ranking are shown here. The bars with * correspond to kmers, whose absence is linked with increased permissiveness. Only orders that were enriched for the kmer are shown.

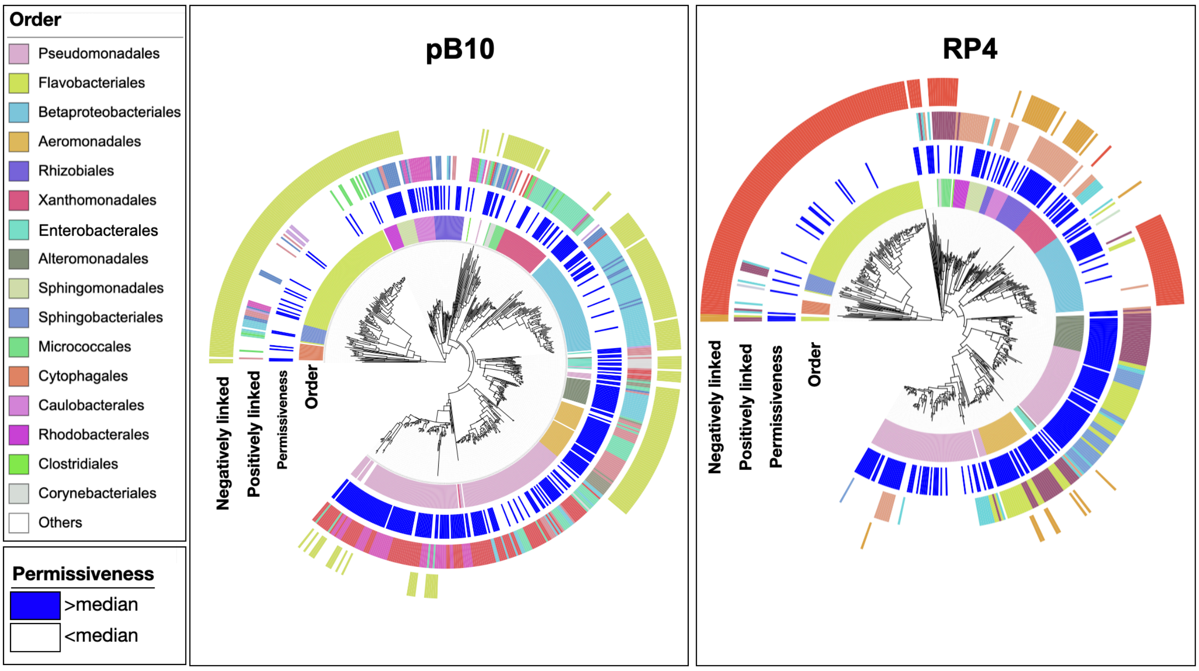

Supplementary Figure S6. The taxa distribution of kmers in Figure S5, which were positively or negatively linked with permissiveness across the tree. The trees were constructed from distances in the kmer profile for taxa's 16S rRNA data. We removed 5 and 11 unannotated taxa from the tree for RP4 and pB10 to improve presentation, respectively. The colors in the positively and negatively linked bands refer to distinct kmers. To improve presentation, only the results for the top ten percent of the kmers, based on their ranking are shown here.

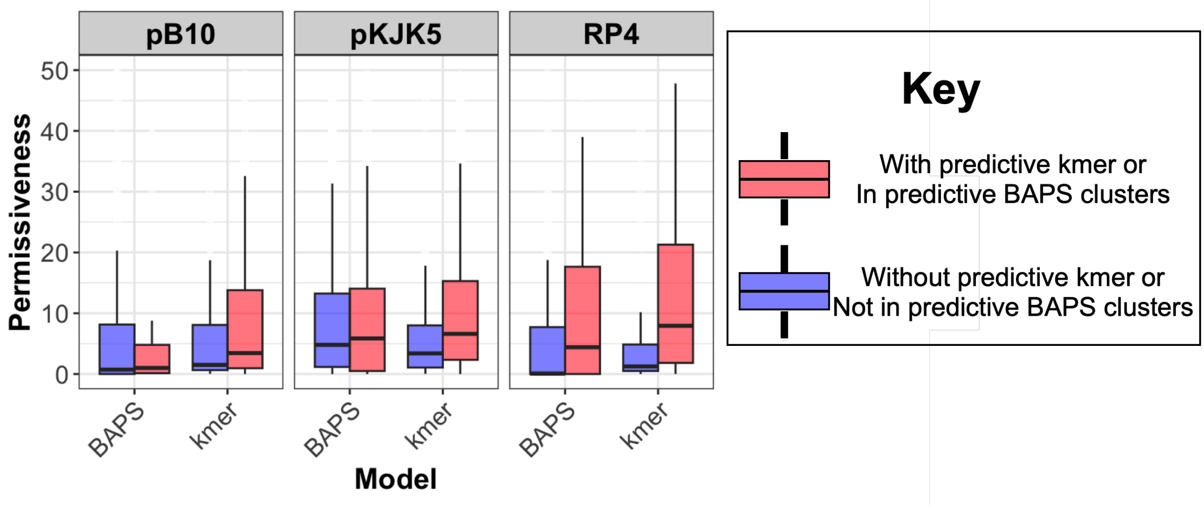

Supplementary Figure S7. The comparison between the permissiveness values for ASVs within (red boxes for BAPS) and outside (blue boxes for BAPS) the predictive BAPS clusters and with (red boxes for kmer) and without (blue boxes for kmer) the predictive kmers for the three plasmids.

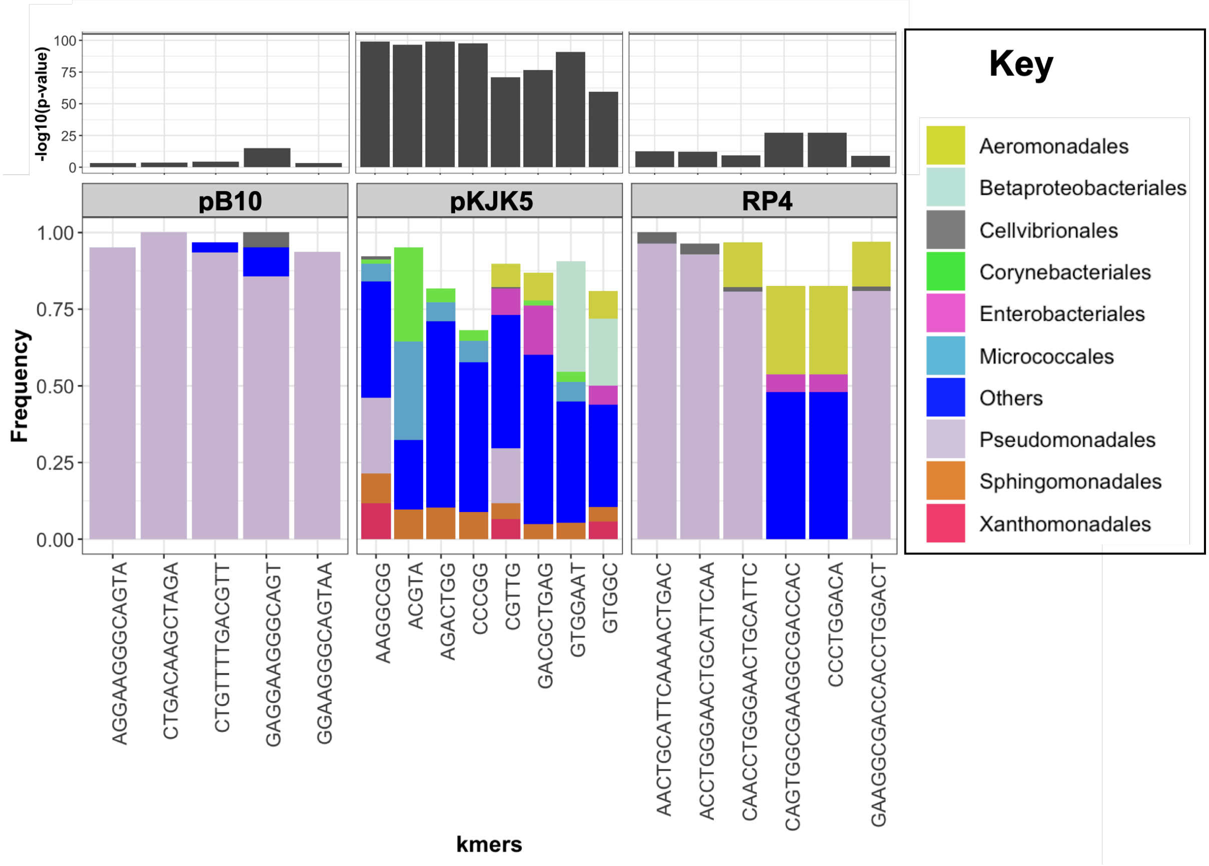

Supplementary Figure S8. Association analysis for significant kmers that were found after accounting for lineage association. Only orders that were overrepresented in the data, i.e., had a higher frequency compared to the baseline frequency in the entire dataset, are shown. The p-value corresponds to the p-value from the association analysis of Scoary, in which the population structure is accounted for.

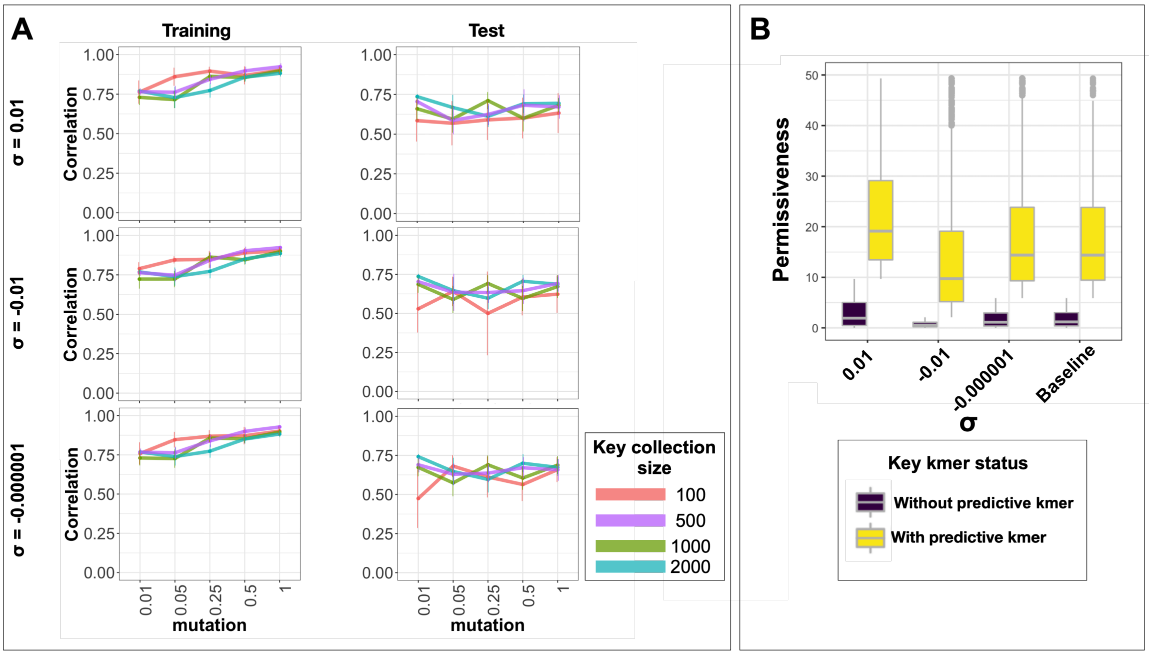

Supplementary Figure S9. The effect of parameters on model performance in simulated datasets. **(A)** The effect of (i) the number of 16S rRNA sequences used for training (population or collection size), (ii) mutation rates and (iii) strength of selection (σ) on the prediction accuracy, using simulated datasets. Each datapoint corresponds to the average of five simulated populations with the specified population size, mutation rate and value for σ (sigma, strength of selection). The error bars show 95% confidence intervals. **(B)** The boxplot shows the permissiveness values for ASVs without and with the predictive kmer with different *σ*, which corresponds to permissiveness for the strains bearing the predictive kmer, respectively. The baseline data shows the permissiveness distribution for pKJK5.

Supplemental Text: Modeling of plasmid transfer in filter mating assays to assess potential plasmid donor limitations to the completeness of transfer

In the filter mating assays to measure the permissiveness of a community of recipients, it is important to ensure that plasmid donors are not limiting the transfer to recipients. We have applied the previous work of Lagido et al. (2003) [1] to assess this.

Filter mating assays, for example, the permissiveness assays, are designed based on calculating the time it takes from initial densities of recipients and donors to form a confluent lawn of bacteria on the filter, assuming this will suffice for all recipients to be sufficiently near a donor to receive a plasmid. For example, Klümper et al. (2014) state that *"The final recipient bacterial concentration is chosen so that assuming an average bacterial size of 1 μm^3 every bacterium will be in contact with the bacterium next to it after four doublings"*[2].

This approximation does not consider that random initial placement of cells tends to result in some large areas with a few cells only, suggesting that it could take considerably longer to reach a confluent lawn and that some recipients could be too far away from donors to come into contact. Therefore, one should calculate how long it would take for all or almost all recipient colonies to grow close enough to a donor colony.

Lagido et al. (2003) have modeled plasmid transfer between colonies growing on agar plates by working out the probabilities of a randomly placed colony being near other colonies and considering how this changes over time when the colony radii expand. A brief summary of this model can be found in [3]. This model of Lagido et al. (2003) directly applies to permissiveness assays and other filter matings. Here we show that in the permissiveness assay carried out by Li et al. (2018) [4], who incubate the filters for 48 hours at 25°C, almost all recipients are predicted to have received a plasmid, if they are permissive, within 14 hours (Figures S10 and S11). This is only somewhat longer than the four doublings required if the cells were equidistantly rather than randomly placed (8.88 h). These times were calculated based on an assumed doubling time of 2.22 h, which may be wrong.

In conclusion, it takes longer for all recipients to get into contact with a donor assuming random rather than equidistant placement, but the incubation time in Li et al. (2018) [4] was very likely sufficient.

Lagido et al. (2003) [1] model assumptions:

- Initially, cells are distributed randomly on the agar or filter surface.
- Each cell grows to form a circular colony. The radius of the colony increases exponentially (colony radius growth rate: g_r). The number of cells increases exponentially too (cell number growth rate: g_n) as long as there are enough resources (nutrients in excess).
- Any plasmid fitness costs are negligible (g_n and g_r are independent of plasmid absence/presence).
- When colonies of donors or transconjugants come into contact with colonies of recipient cells, all the recipient cells become transconjugants after a conjugation time t_{conj} (=0). This simplification is probably the most problematic assumption.

- Plasmid loss is negligible over the time simulated (the plasmid is stable)
- Cells or colonies do not move other than expand by growth

Table 1: Parameters

Symbol	Meaning
$N_{D,0}$	Initial number of donor cells
$N_{R,0}$	Initial number of recipient cells
A	Area of the membrane filter
g_r	Colony radius specific growth rate
r_0	Initial colony radius
g_n	Cell number specific growth rate
λ	$(N_{D,0} + N_{R,0})/A$ (areal density)

Equations from Lagido et al. (2003)

Probability that a colony is in a clump of n colonies after time t

$$p_n(t) = \exp(-4r_0^2\pi\,\lambda e^{2g_rt})(1 - \exp(-4r_0^2\pi\,\lambda e^{2g_rt}))^{n-1}$$

Probability that a colony is a recipient after time t

$$\frac{p_1(t)\,N_{R,0}}{N_{D,0} + p_1(t)N_{R,0}}$$

Number of donor ($N_{D,t}$), number of recipient ($N_{R,t}$) and number of transconjugant ($N_{T,t}$) cells after time t ($t \leq t_{max} = (\ln(N_{max}) - \ln(N_{D,t} + N_{R,t}))/g_n$)

$$N_{D,t} = N_{D,0}e^{g_nt}$$

$$N_{R,t} = \frac{(N_{D,0} + N_{R,0})p_1N_{R,0}e^{g_nt}}{N_{D,0} + p_1N_{R,0}}$$

$$N_{T,t} = N_{R,0}e^{g_nt} - N_{R,t}$$

Parameter values corresponding to the experiments in Li et al. (2018)
$N_{D,0} = 3 \cdot 10^7$ cells
$N_{R,0} = 3 \cdot 10^7$ cells
$A = \pi r^2 = \pi(25{,}000\,\mu m)^2 = 1.96 \cdot 10^9\,\mu m^2$
$r_0 = 0.8\,\mu m$

Relationship between cell and colony radius growth rates

$g_n = 0.3125\ h^{-1} \Rightarrow$ Time of doubling: $t_d = \frac{\log(2)}{g_n} = 2.22\ h$

$g_r = 0.5 \cdot g_n$

Justification: we assume that the colony is a monolayer (or has constant height).
Hence, the area of the colony is proportional to (symbol ∝) the number of cells, and the number of cells increases exponentially with growth rate g_n:

Colony area $\propto$ number of cells $\propto e^{g_n t}$ (1)

Also, the area is proportional to the square of the radius of the colony and that radius increases exponentially with growth rate g_r:

Colony radius $\propto \sqrt{\text{Colony area}}$ Colony radius $\propto e^{g_r t}$ (2)

Then, since we have to satisfy both (1) and (2):

$$\left.\begin{array}{l}\text{Colony radius} \propto e^{g_r t} \\ \text{Colony radius} \propto \sqrt{e^{g_n t}}\end{array}\right\} \Rightarrow g_r = 0.5 \cdot g_n$$

NB: If the proportionalities (1) and (2) were rewritten as equivalent equations (i.e., introducing proportionality constants), the relationship $g_r = 0.5 \cdot g_n$ would include a constant that would vanish with time, converging on the above relationship.

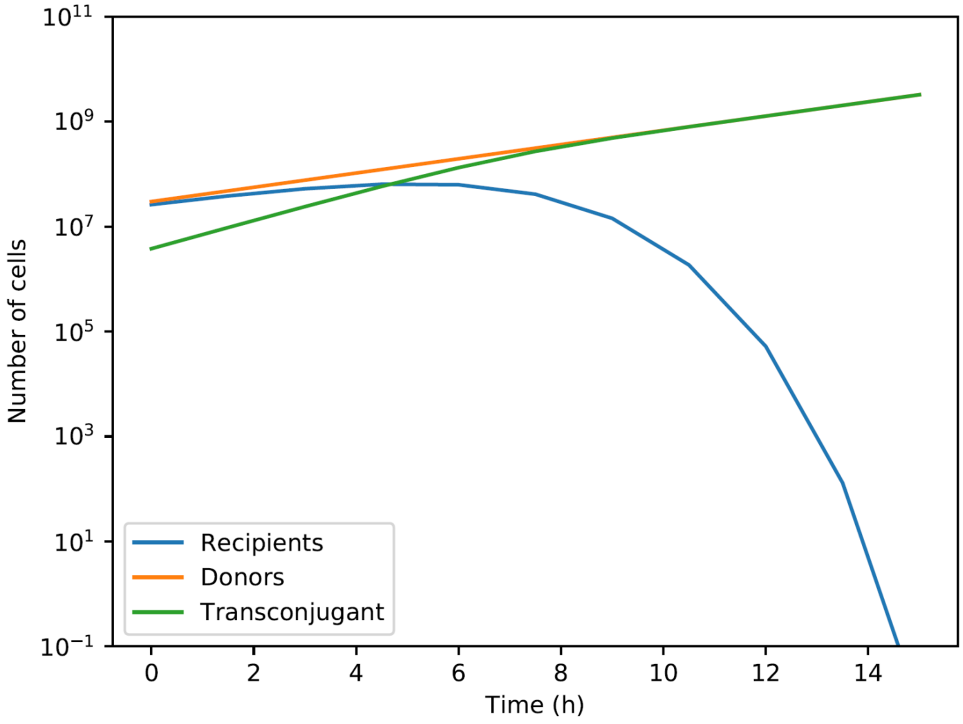

Supplementary Figure S10. Predicted change of the number of transconjugant, donor and recipient cells over time.

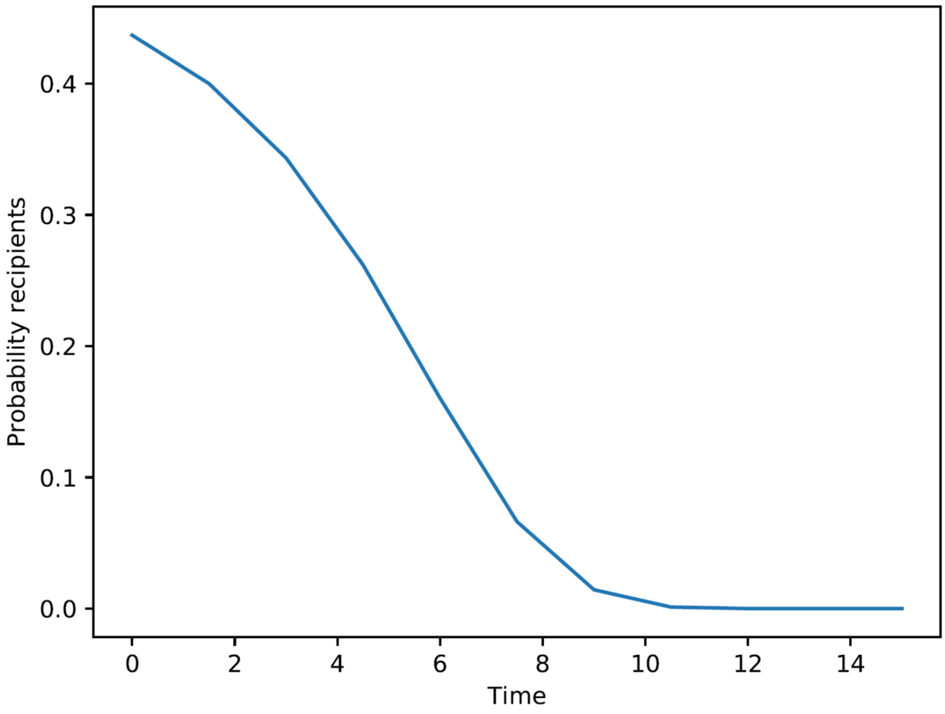

Supplementary Figure S11. Probability that any colony is still a recipient.

References

1. Lagido, C., et al., *A model for bacterial conjugal gene transfer on solid surfaces.* FEMS Microbiol Ecol, 2003. **44**(1): p. 67-78.
2. Klümper, U, Dechesne A, Smets B.F., *Protocol for Evaluating the Permissiveness of Bacterial Communities Toward Conjugal Plasmids by Quantification and Isolation of Transconjugants.* Hydrocarbon and Lipid Microbiology Protocols, 2014.
3. Kreft, J.-U., *Mathematical modelling of plasmid dynamics.*, in *Molecular Life Sciences: An Encyclopedic Reference.* . 2014, Springer-Verlag, Berlin Heidelberg.
4. Li, L., Dechesne, A., He, Z., Madsen, J.S., Nesme, J., Sørensen, S.J., Smets, B.F., *Estimating the Transfer Range of Plasmids Encoding Antimicrobial Resistance in a Wastewater Treatment Plant Microbial Community.* Environ. Sci. Technol. Lett. , 2018.
